# The KRAB Domain of ZNF10 Guides the Identification of Specific Amino Acids That Transform the Ancestral KRAB-A-Related Domain Present in Human PRDM9 into a Canonical Modern KRAB-A Domain

**DOI:** 10.3390/ijms23031072

**Published:** 2022-01-19

**Authors:** Peter Lorenz, Felix Steinbeck, Ludwig Krause, Hans-Jürgen Thiesen

**Affiliations:** 1 Rostock University Medical Center, Institute of Immunology, Schillingallee 70, 18057 Rostock, Germany; felix.steinbeck@uni-rostock.de (F.S.); ludwigkrause@t-online.de (L.K.); hj.thiesen@gmx.de (H.-J.T.); 2 Gesellschaft für Individualisierte Medizin (IndyMed) mbH, 17, 18055 Rostock, Germany

**Keywords:** KRAB, PRDM9, ZNF10/KOX1, SSX, TRIM28, HAP1 cells, transcription, repression, *Latimeria chalumnae*, coelacanth, AlphaFold2, CRISPRi

## Abstract

Krüppel-associated box (KRAB) zinc finger proteins are a large class of tetrapod transcription factors that usually exert transcriptional repression through recruitment of TRIM28/KAP1. The evolutionary root of modern KRAB domains (mKRAB) can be traced back to an ancestral motif (aKRAB) that occurs even in invertebrates. Here, we first stratified three subgroups of aKRAB sequences from the animal kingdom (PRDM9, SSX and coelacanth KZNF families) and defined ancestral subdomains for KRAB-A and KRAB-B. Using human ZNF10 mKRAB-AB as blueprints for function, we then identified the necessary amino acid changes that transform the inactive aKRAB-A of human PRDM9 into an mKRAB domain capable of mediating silencing and complexing TRIM28/KAP1 in human cells when employed as a hybrid with ZNF10-B. Full gain of function required replacement of residues KR by the conserved motif MLE (positionsA32-A34), which inserted an additional residue, and exchange of A9/S for F, A20/M for L, and A27/R for V. AlphaFold2 modelling documented an evolutionary conserved L-shaped body of two α-helices in all KRAB domains. It is transformed into a characteristic spatial arrangement typical for mKRAB-AB upon the amino acid replacements and in conjunction with a third helix supplied by mKRAB-B. Side-chains pointing outward from the core KRAB 3D structure may reveal a protein-protein interaction code enabling graded binding of TRIM28 to different KRAB domains. Our data provide basic insights into structure-function relationships and emulate transitions of KRAB during evolution.

## 1. Introduction

DNA-binding transcription factors are key elements of control in converting a genotype into the desired phenotype of a cell [1,2]. Consequently, the study of their mechanisms of action and complex regulatory behavior is central to understanding all biological programs relying on transcription. Constitutive and facultative transcriptional silencing plays an important role in establishing and regulating cellular identities at the level of chromatin organization [3,4]. Initially, DNA-binding by selective transcription factors specifies the genomic loci to recruit protein complexes that reorganize chromatin into a state non-permissive for transcription [5,6]. These protein complexes include corepressor and modulator protein assemblies that mediate DNA methylation, catalyze specific histone post-translational modifications and effect chromatin remodeling [7]. An example of such mechanisms is the RE1 silencing transcription factor (REST/NRSF), which assembles differing repressor complexes depending on cell type and biological context to restrict the transcription of target genes [8].

The most abundant class of transcription factors in tetrapods is characterized by a DNA-binding motif called the C2H2 zinc finger (ZNF). In humans, this class has more than 700 members [1,2]. About half of those contain an N-terminal protein-protein interaction domain called the Krüppel-associated box (KRAB), giving this subclass the designation KRAB zinc finger (KZNF) proteins. First discovered as a “heptad repeat of leucines” in ZNF10/Kox1 [9], the KRAB domain was quickly discovered in many ZNF proteins in all tetrapod lineages with a particular expansion by gene duplication in mammals [10,11,12]. It has been proposed that such proteins evolved in the common ancestor of lobe-finned fish and tetrapods [11]. Thus, this transcription factor family evolved during the time of the major evolutionary step for vertebrate organisms from living in water to living on land.

However, sequences with significant homology to KRAB can also be traced in invertebrates leading to the postulation of more ancestral KRAB sequences [13]. Those sequences are not only found in evolutionary old invertebrate species such as sea urchin, but their homologs still exist in mammals, including humans, in the PR/SET domain 9 (PRDM9) and synovial sarcoma X breakpoint (SSX) ortholog and paralog groups [13,14]. In this publication, we refer to these more ancient KRAB domains as ancestral KRAB (abbreviated aKRAB) in contrast to the modern KRAB (mKRAB). 

The classical mKRAB domains of canonical function are in general characterized by two interdependent properties. They confer transcriptional repression in heterologous reporter assays and form a complex with the chromatin modulator protein tripartite motif containing 28 (TRIM28, synonyms: SMP1, KAP1, TIF1beta) [15,16,17]. The canonical mechanisms of transrepressor activity involve recruitment of histone deacetylase and methyltransferase (in particular SETDB1) activities and heterochromatin proteins with TRIM28 acting as a hub [16,18,19]. KZNF proteins, often in concert with TRIM28, have been shown to participate in many biological processes, including cell proliferation, differentiation, development, imprinting, metabolism and pathophysiology [16,17]. With respect to cancer, KZNF proteins, as well as TRIM28 and SETDB1, have been shown to exert pleiotropic roles as oncogenes or tumor suppressors in different tumor types [20,21,22]. The ongoing evolutionary contention between host and retroviruses is considered a major source for expansion and emergence of new members of KZFN genes in species of different phylogenetic branches [23,24]. Of major interest is the role of KZNF proteins in the restriction of retroviral genomic sequences, in particular during development, and for exaptation, i.e., their repurposing for novel regulatory principles [16,25,26]. In conjunction with CRISPR/Cas the ZNF10-KRAB is used as a tool for modulating and redirecting transcriptional gene regulation in basic research (CRISPRi; [27,28]), and applied in oncology (e.g., [29,30]), in human genetics (e.g., [31]) and virology (e.g., [32]).

KRAB domains containing up to about 75 amino acids are modular and can exist in different configurations with respect to subdomain composition. In general, the N-terminal KRAB-A domain is indispensable for transcriptional repressor activity and interaction with other proteins, in particular TRIM28. However, while some A subdomains are functionally self-sufficient, others rely on a second C-terminal auxiliary subdomain called KRAB-B for full activity or function [33,34,35]. In addition, KRAB-B subdomains exist in different “flavors” called B, b, BL and C [36]. It is currently unclear which sequence determinants in KRAB-A render it dependent or independent of a KRAB-B subdomain. 

PRDM9 is a meiosis-specific protein which serves as a pioneering factor for determining the sites of meiotic recombination events [37,38]. It contains a KRAB domain of the ancestral type followed by a synovial sarcoma, X breakpoint repression domain (SSXRD; [39]), a PR/SET domain with histone H3 lysine 4 and lysine 36 trimethylation activity and tandem repeats of C2H2 zinc fingers for DNA binding. The zinc finger domains have been shown to rapidly evolve, a phenomenon thought to contribute to speciation [40,41,42]. While many vertebrate species contain PRDM9 orthologs encompassing this whole domain configuration, some phylogenetic branches have lost some domains or are even completely devoid of PRDM9 genes, such as the amphibians, birds and crocodiles [14]. However, only PRDM9 genes encoding the ancestral KRAB and SSXRD configuration together appear to display the rapid evolution of zinc fingers [14]. Importantly, in contrast to a canonical KRAB, the aKRAB of human PRDM9 neither silences transcription nor interacts with TRIM28 [33,43,44].

The SSX family of proteins consists of a number of paralogs in most mammalian species. In humans, at least nine complete genes have been described with additional pseudogenes [45]. The encoded proteins are similarly configured as the PRDM9 N-terminal part and comprise an N-terminal aKRAB-A and a C-terminal SSXRD domain. SSX proteins belong to the functional class of cancer-testis antigens since they are aberrantly expressed in cancer and can contribute to formation of oncogenic gene fusions via chromosomal rearrangements in tumor cell genomes [46,47]. Recent work implied functional connections of SSX proteins to Polycomb complexes at pericentromeric heterochromatin [46]. While the aKRAB-A domains of SSX1 and SSX2 only confer very weak repressor activity compared to the canonical mKRAB of ZNF10, most potential for transcriptional repression resides in SSXRD [39].

Here, we wanted to investigate how many, and which, sequence changes had to occur to transform an ancient KRAB-A-like domain that neither interacts with TRIM28 nor confers transcriptional repression into a canonical KRAB domain which displays those properties. Such changes might re-enact necessary steps evolution has taken to develop the modern KRAB domain as a recruiter of repressor complexes built around the hub protein TRIM28. Our approach started with dedicated mutant aKRAB constructs of human PRDM9 guided by the analysis of sequence conservation and previous knowledge on the mKRAB of human ZNF10. Mutants were evaluated in transcriptional reporter and protein-protein complex formation assays, as well as through 3D modelling by AlphaFold2. Our results showed a similar core structure made of α-helices for the ancestral as well as the modern KRAB. However, gain of function of PRDM9 aKRAB required a specific insertion and several replacements of residues at specific positions. The changes reoriented part of the side-chains of the central α-helix and provided novel side-chains with different physicochemical properties. Importantly, the amino acid changes only led to function in concert with the ZNF10-B domain that appears to be involved in forming the characteristic spatial layout of modern KRAB-AB domains. Our structure-function-analysis of aKRAB/mKRAB mutants describes a potential evolutionary path that occurred from sarcopterygian fish to tetrapods, and thus from water to land-living vertebrates that established novel functional repressor mechanisms of transcriptional regulation.

## 2. Results

### 2.1. Consolidation of the Ancestral KRAB Domain (aKRAB)

**Figure 1:**
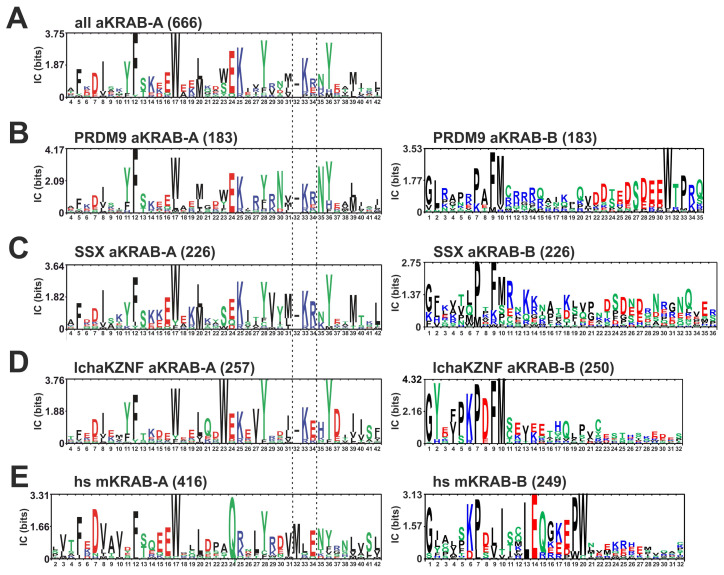
Sequence logos describing the conservation within the different groups for the complete ancestral KRAB group (aKRAB; panel **A**), the PRDM9 ortholog subgroup (**B**), the SSX ortholog/paralog subgroup (**C**), the *Latimeria chalumnae* KRAB zinc finger subgroup (lchaKZNF; **D**) and the modern KRAB represented by human sequences (hs mKRAB-A; **E**). The left side logos reflect the KRAB-A sequences, the ones to the right KRAB-B. Residue numbers below each logo reflect amino acid position. Numbers for mKRAB-A coincide with coding exon codon number. In this manuscript we numbered the residues according to KRAB subdomain starting from 1 for each subdomain, followed by position and one letter symbol. The B domain sequences are consecutive to KRAB-A in the full proteins. Numbers in round brackets denote the number of sequences of the group that contributed to a logo. The two vertical broken lines at positions A32-34 highlight the three-amino acid region conserved in mKRAB-A which contain a gap in aKRAB-A sequences (dash in respective logos). The y-axis denotes information contents (bits). Note that we treat the gap in alignment for aKRAB-A like an amino acid to make position numbering easier to compare to mKRAB-A. Full protein sequences with their associated aKRAB-A sequences and assignment to the subgroups are provided in Table S1. Here and throughout the manuscript amino acids with nonpolar side-chains are colored black, those with polar side-chains are in green, ones with basic side-chains are blue and those with acidic side-chains are red.

**Figure 2:**
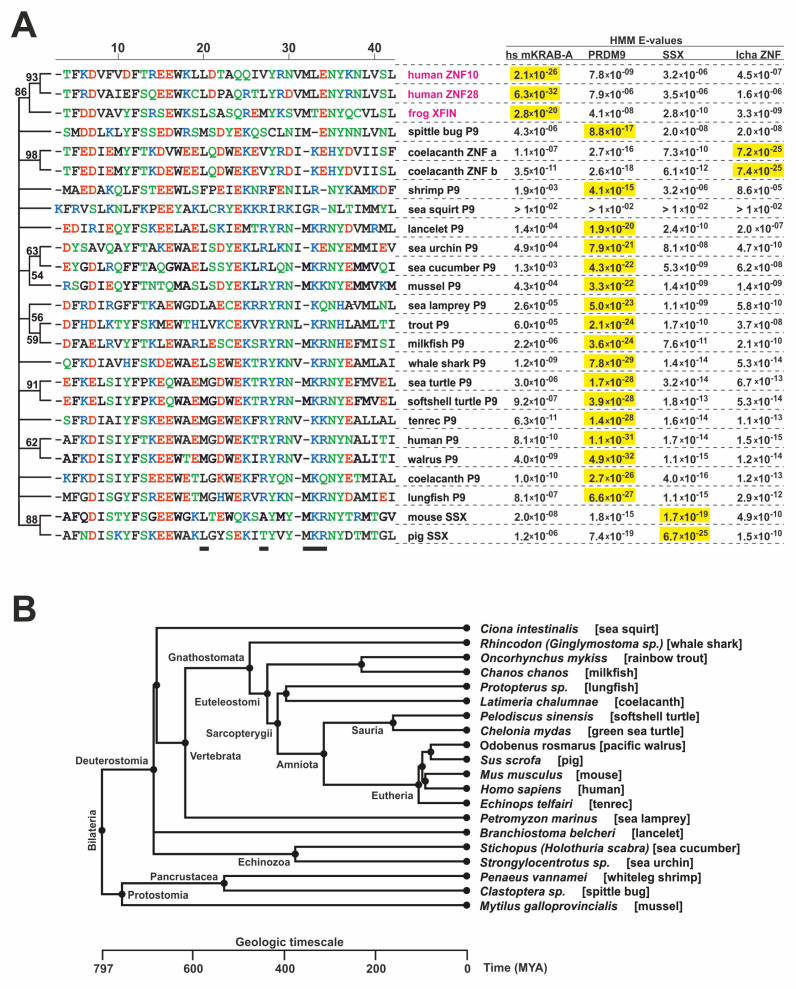
Evolutionary snapshot of the ancestral KRAB-A sequences. (**A**). Depiction of selected KRAB-A sequences (ClustalW alignment, manually curated around positions 30–32) along with the HMM scores (E-values; the lower, the better) against the different KRAB-A models. Selection based on best hits of a group member vs the different KRAB-A models, inclusion of phylogenetically oldest species and added outliers (names in red). Outliers are ZNF10-A as the “gold standard” of a canonical KRAB-A (transcriptional repression through TRIM28 recruitment), ZNF28-A as one of nine identical human KRAB-A sequences with best score against the hs mKRAB HMM and XFIN-A, a proven canonical KRAB-A from the evolutionary oldest class of tetrapods (amphibians; [33]). The aligned sequences are ordered according to a Maximum Likelihood consensus tree (see Methods Section 4.2). The bootstrap consensus tree derived from 1000 replicates. Only stable clusters with >50% percentage of coinciding replicate trees are labeled, the unstable ones are collapsed. (**B**). Timetree (www.timetree.org accessed on 25 August 2021) built on the species designations from the sequences included in part A (*Xenopus* omitted since amphibians do not contain aKRAB sequences; see text). If a particular species was missing in the database a close other species was selected (e.g., same *genus;* abbreviation “sp.” in tree or replacing species given in round brackets). Branching points are labeled according to Timetree annotation. Note that *Branchiostoma* and the two *Echinozoa* species are not resolved in the tree although only the former belongs to *Chordata*.

First, we generated a comprehensive catalog of KRAB-A domains that reflect the evolutionary older sequence configuration that has been described for PRDM9 KRAB-A from humans and its ancestral ortholog in the sea urchin, as well as in human SSX proteins [13]. We refer to such KRAB-A domains as the ancestral KRAB-A class, abbreviated aKRAB-A, in contrast to modern KRAB-A, designated mKRAB-A. To date most publications and databases do not make this distinction. However, the InterPro protein resource already contained a compilation of aKRAB-A sequences under the header “Krüppel-associated box-related” (InterPro domain IPR003655; InterPro version 77.0, November 2019). Inspection of this list revealed that it contained mostly sequences with the status “unreviewed” and included a substantial number of sequences from bacteria and fungi. The latter sequences show only low homologies to aKRAB-A of human PRDM9 and SSX1 and do not score with E-values < 0.01 against our initial profile Hidden Markov Model (HMM) of aKRAB-A using hmmscan. Therefore, we curated the InterPro entries using profile HMMs as well as BLAST approaches (see Methods). We also included the recently described KRAB-A sequences from the coelacanth *Latimeria chalumnae* (abbreviation “lcha”) KRAB-ZNF proteins [11] that fit the aKRAB-A type much better than canonical KRAB-A sequences like the one from human ZNF10/KOX1 [48]. Our final compilation contained 666 entries from 155 species (Table S1, sequence logos in Figure 1). They all belong to *Bilateria* and the entries consist of PRDM9 and SSX orthologs/paralogs and the coelancanth aKRAB-A KZNF proteins. We did not find any aKRAB sequences in amphibians and birds. The lack of PRDM9 orthologs in these animal classes has been described previously [14]. In addition, all SSX orthologs/paralogs were restricted to mammals. Specific profile HMMs clearly distinguished the PRDM9, SSX and lchaZNF subgroups with high confidence (Table 1 and Table 2). The strong separation of the aKRAB-A subgroups from the human KRAB-A subfamily, representing mKRAB-A domains, was strongly evident in the largest differences in the scores. When looking at individual members of a group, each subgroup member scored best with its own profile HMM compared to the other subgroups’ profile HMMs using HMMER (see individual HMM E-values given in Table S1 and the selected examples in Figure 2). Thus, our profile HMMs for aKRAB-A and subgroups will be useful in classifying existing and novel KRAB domain proteins in sequence databases. In terms of evolutionary age, the members of the aKRAB-A family were evident not only in *Deuterostomia*, including the vertebrates, but also contained members from *Protostomia* such as insects and mussels (see compilation in Table S1 and the selected examples in Figure 2). This argues that the first aKRAB-A sequences existed at least 700 million years ago (MYA) according to a species time tree (www.timetree.org accessed on 25 August 2021; [49]; Figure 2). 

The consensus sequence logo of the aKRAB-A domain defined by its profile HMM contains 38 amino acids (Figure 1A). The comparison to the logo of the human mKRAB-A sequences (Figure 1E) revealed a number of similarities, but also obvious differences. Most strikingly, the logo alignment illustrates a telltale gap in aKRAB-A in the region of the conserved motif VMLE (positions KRAB-A31-34) of canonical KRAB domains. Instead, aKRAB-A most often harbors a three-residue stretch of M/I/L/V/gap/KR. Mutation of the MLE motif has been found to abrogate canonical KRAB-mediated transcriptional repression and TRIM28 binding [50,51,52,53] as did insertion of double proline residues as helix breaker before E34 [33,54]. Therefore, we hypothesized that differences in spacing and in side-chain properties most likely contribute to functional differences between ancestral and modern KRAB families. Another charge difference exists at residue A24 (most prevalent amino acid E) of aKRAB-A compared to human mKRAB-A (almost always Q). Further, the highly conserved DV part at position A7-8 of human mKRAB-A usually reads DI in aKRAB-A. Differences are also seen between aKRAB-A subgroups (Figure 1B–D). Interestingly, the coelacanth KRAB-ZNF proteins have a higher incidence of E or D at position A34 (Figure 1D) compared to R/E and R/K of the PRDM9 and SSX aKRAB-A subgroups (Figure 1B,C). Thus, it looks like one basic residue has already switched towards the A33-34/LE sequence of mKRAB in the MLE motif in this species during evolution. Another example is the much higher frequency of A23/W of the coelacanth compared to the other two aKRAB-A subgroups.

Conserved amino acid sequences specifying the mKRAB-B subdomain follow mKRAB-A domains in a considerable number of cases (see introduction). Among the proteins with aKRAB-A, we only found some coelacanth KZNF proteins that score with the HMM matrix of human KRAB-B at all, although only with high E-values, i.e. low scores (data not shown). However, we noticed that aKRAB-A domains appear to also have conserved amino acid patterns in the residues subsequent to the A subdomain. We therefore made alignments and constructed logos for those protein stretches as well. The results indicated that the sequence region following aKRAB-A is most often characterized by the motif PxxFM at positions B7 to B10 in what we decided to name ancestral KRAB-B (aKRAB-B; Figure 1, logos to the right). The aKRAB-A subgroups were also distinguished by their subsequent aKRAB-B sequences. While the PRDM9 ortholog group has a higher frequency of B10/M compared to the SSX group and the strongly conserved residue B31/W, the coelacanth aKRAB-B paralogs are highlighted by a longer motif of B5-10/PKPDFM. Interestingly, residues B6-8/KPD are a characteristic of the modern KRAB-B. However, there, these three amino acid residues are usually accompanied by B13-14/LE and B19-20/PW. Altogether, the observed sequence conservation motifs suggest a scenario in which more ancestral KRAB domains evolved from very ancient PRDM9 members at the base of *Protostomia* to premodern KRAB in the ancestor of the coelacanth and finally to modern KRAB in tetrapods. Characteristic patterns of linked amino acid residue only reflect the assignment to specific subgroups but surely also influence physicochemical and associated biological properties. Phylogenetic sequence changes are likely to have resulted in transitions in protein interaction partners that may cause functional differences in the KRAB domain superfamily and may contribute to subspecies formation.

### 2.2. Transformation of PRDM9 aKRAB-A into a Modern Canonical KRAB

We decided to establish a roadmap of how an evolutionary older aKRAB-A may have been transformed into a modern KRAB-A domain that displays canonical functions, i.e., it confers transcriptional repression and forms a complex with TRIM28. As starting point we selected the aKRAB-A of human PRDM9. As blueprint for a canonical mKRAB-A, we chose the one from human ZNF10. The ZNF10 KRAB was the first KRAB domain to be identified and has been instrumental in analyzing KRAB-mediated transcriptional repression and identifying TRIM28 as a major KRAB-interacting protein [9,15,51,55,56]. Since the ZNF10 mKRAB-A only functions efficiently when teamed up with its cognate KRAB-B [33,35], it was essential to add authentic ZNF10 KRAB-B sequences to the aKRAB-A mutants to be tested. 

### 2.3. Heterologous Reporter Assay for Transcriptional Repression

**Figure 3:**
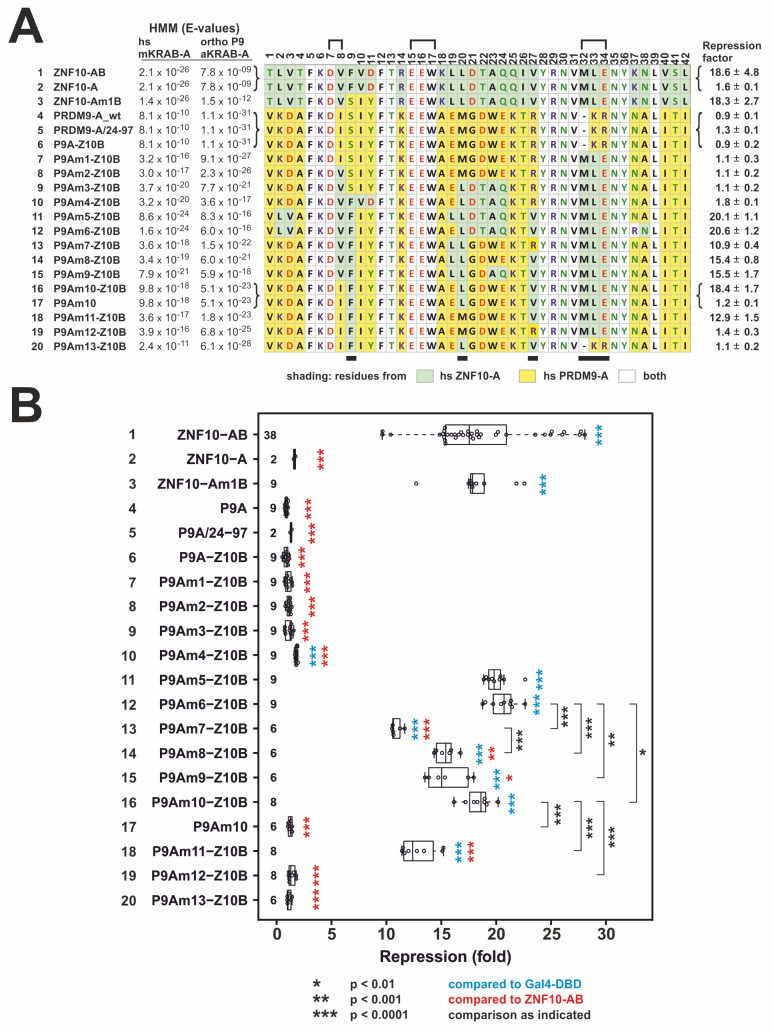
Alignments of examined KRAB-A sequences and results of heterologous reporter assays for transcriptional repression of respective constructs. (**A**). Numbered rows with designation of the respective construct made as fusion protein with the Gal4 DNA binding domain (Gal4-DBD). The PRDM9 aKRAB-A mutants (denoted as P9Am with a specific number) were usually hooked up to a ZNF10-B subdomain (denoted as Z10B). Some constructs do not differ in KRAB-A (curved brackets) but with respect to KRAB-B which can be either missing, be authentic PRDM9/65-97 or ZNF10-B sequence (B not shown in this alignment). The respective aligned KRAB-A sequence of each construct was colored for amino acid properties (single letter code color: red = acidic; blue = basic; green = polar; black = nonpolar) and match to ZNF10-A or PRDM9-A (shading; see legend below alignment). HMMer score E-values (smaller = better) of each KRAB-A against the human mKRAB-A and the PRDM9 ortholog group aKRAB-A are given to the left of each sequence. The mean repression factor ± standard deviation for each construct based on reporter assays for transcriptional repression in HeLa cells is shown on the right. Square brackets above the numbering of the alignment denote amino acid groups whose mutations have been shown to disrupt transcriptional repression of ZNF10-AB [51]. (**B**). Detailed data box plot obtained after testing the different constructs in HeLa cells 24 h post-transfection and using Gal4-DBD as reference set to 1. Numbers to the left of each construct’s name fit the numbers in the A part of the figure. Integers to the left of each box plot indicate the number of data points. For almost all constructs we performed at least six biological replicates from three independent experiments. Results of a two-tailed unpaired t-test are given as asterisks as specified in the legend below the plot.

Here, we used a classical dual luciferase reporter assay for transcriptional repression using fusions of yeast transcription factor Gal4 DNA-binding domain (Gal4-DBD) with the KRAB domain to be analyzed as effector protein (Figure 3). Closeness of ZNF10-A and PRDM9-A to the respective mKRAB-A/aKRAB-A groups is indicated by the better fits (lower E-values) to the group-specific profile hidden Markov models (HMM, as indicated in Figure 3A). The repressive potencies of wild-type PRDM9 as an isolated aKRAB-A, a fusion with ZNF10-B and an extended aKRAB-A (PRDM9/AA-24-97) all were nonexistent or negligible (0.9; 0.9, 1.3-fold over Gal4-DBD alone; lines 4–6 in Figure 3A,B). These results coincided with our previous analysis of transcriptional repression of the N-terminal PRDM9 domains [33]. The end point of the analysis for potent repression, the canonical mKRAB-A of ZNF10 together with its authentic B domain had mean values around 19-fold. 

The first PRDM9 aKRAB-A mutant, P9Am1, was characterized by the introduction of the conserved MLE residues (positions 32–34) instead of the gap-KR sequence of wild-type PRDM9-A. Thus, the telltale alignment gap when comparing aKRAB-A and canonical mKRAB-A domains was closed and two positive residues were replaced with two hydrophobic and one acidic amino acid. Despite these important changes towards an mKRAB-A domain, our reporter assay clearly demonstrated that the P9Am1 mutant was inactive as a transcriptional repressor (Figure 3A,B, line 7). Thus, the structural and charge changes in the VMLE region of KRAB-A were not sufficient to confer canonical transrepressor activity despite our experience in replacing MRG by MLE in ZNF746 [50]. 

Next, we stepwise exchanged original PRDM9 residues for the amino acids occurring at the respective positions in ZNF10-A. The first mutant to reach repression activity of the same potency as ZNF10-AB was P9Am5-Z10B (Figure 3A,B, line 11). This mutant had altogether 13 amino acid changes. To pinpoint the important residues in KRAB-A for gain of activity, we then worked back from this mutant by reintroducing original PRDM9 amino acids at respective positions. The mutant with the least changes from PRDM9-A wild-type to ZNF10-A, but with full ZNF10-AB-like repressor potency of around 18-fold, was the P9Am10 configuration (Figure 3A,B, line 16). P9Am10 had only three further replacements at specific positions in addition to the MLE insertion compared to wild-type PRDM9-A. These were F9 instead of S9, L20 for the original M20 and V27 took the place of R27. Further experiments with changes at specific positions and the analysis of a ZNF10-Am1 mutant (amino acids 10–12 switched from ZNF10-A FVD to the PRDM9 original SIY) led to the following conclusions. The ZNF10-A-derived F9 residue is very likely not of importance for the potent activity of P9Am10-Z10B KRAB since the ZNF10Am1B mutant with this residue is as potent in repression as the wild-type ZNF10-AB (Figure 3A,B, line 3 vs. 1). In contrast, gain in activity from the P9Am1 to the P9Am10 mutant KRAB was most strongly determined by the presence of the hydrophobic L20 and V27 residues together. Indeed, consecutive switching back of L20 to original PRMD9 M20 and V27 to R27 dropped repressor activity from around 18-fold first down to about 13-fold (in mutant P9Am11-Z10B, Figure 3A,B, line 18) and then to only 1.4-fold (P9Am12-Z10B, Figure 3A,B, line 19). V27 appears to be more important since P9Am4-Z10B displayed only a borderline 1.8-fold repression activity (Figure 3A,B, line 10) despite having more replacements compared to P9Am10-A, but having the original R27 and lacking V27. The importance of L20 and V27 does not negate the requirement for the MLE insertion. This was indicated with mutant P9Am13-Z10B, in which the P9Am10-A sequence retained the two residues, but the MLE insertion was mutated back to gap-KR. The switchback abolished repression activity completely (Figure 3A,B, line 20). Of further interest is the observation, that the highly conserved valine of the motif DV (residues 7, 8) in canonical KRAB-A can be replaced without loss of repressor activity by isoleucine. Mutant P9Am10 contains this PRDM9 wild-type DI motif, as does the majority of aKRAB-A domains in both PRDM9 and SSX proteins (see logos in Figure 1). In conclusion, gain of canonical repressor activity of the aKRAB-A of PRDM9 required insertion of the conserved MLE sequence for proper positioning and to replace positive charges, and the introduction of L20 and V27, in which the latter replaces a positively charged R residue. 

It is important to note that these observations reflect AB-type KRAB domains. P9Am10 is only a potent repressor if fused to ZNF10-B (compare Figure 3A,B, lines 16 and 17): Omission of ZNF10-B dropped repressor activity from about 18-fold down to 1.2-fold only. Thus, changes of amino acid residues at specific positions in PRDM9-A had to be accompanied by acquisition of B subdomain sequences during evolution, at least when compared to AB-type mKRAB domains.

**Figure 4:**
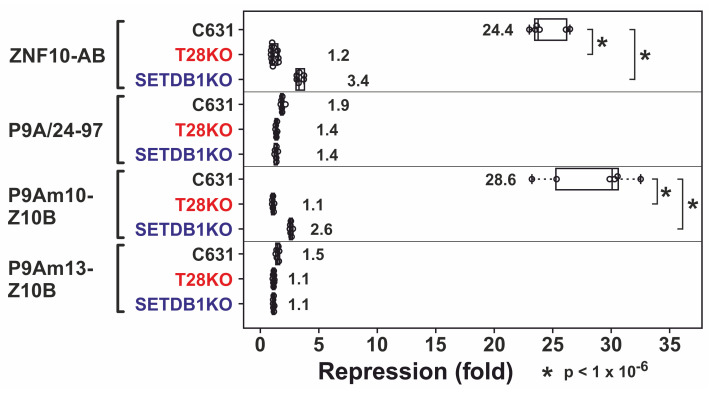
Heterologous luciferase reporter assays in HAP1 wild-type (C631), TRIM28 knockout (T28KO) and SETDB1 knockout (SETDB1KO) cell lines. Box plots depict normalized repression factors against Gal4-DBD alone tested in the same cell line. Numbers in the plot indicate the mean repression factor ± standard deviation for each construct. Data are from at least six biological replicates from at least three independent experiments. The asterisks indicate a *p*-value below 1 × 10^−6^ for the comparisons indicated by the brackets.

Canonical KRAB domains rely on functional TRIM28 and SETDB1 for exerting their repressive activities [50,57,58]. To test whether the P9Am9-Z10B mutant in addition to its similar potency compared to ZNF10-AB meets these requirements as well, we tested its activity in HAP1 TRIM28 knockout and SETDB1 knockout cells (Figure 4). Indeed, P9Am10-Z10B lost its transcriptional repressor function completely in cells devoid of TRIM28. Similarly, in the absence of SETDB1, suppression was largely lost, with some residual activity. Thus, P9Am10-Z10B behaved exactly like the canonical ZNF10-AB. The borderline low and nonexisting repressive potentials of P9A/24-97 wild-type fragment and the P9Am13-Z10B mutant were reproducible in the HAP1 cell lines. 

### 2.4. Complex Formation with TRIM28

**Figure 5:**
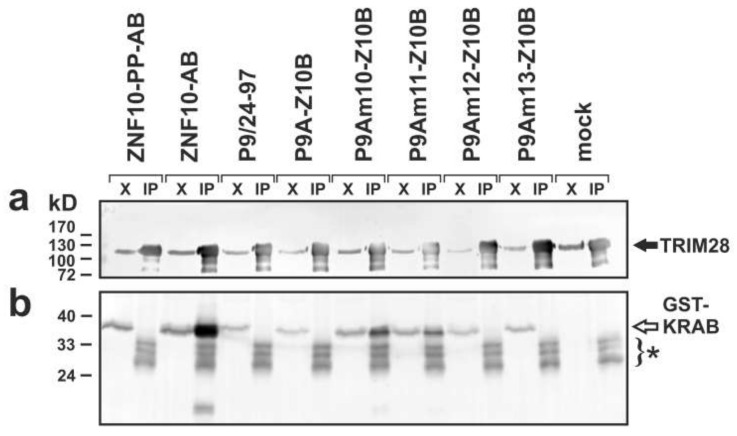
Evaluation of selected KRAB domains for complex formation with cellular TRIM28 by co-immunoprecipitation followed by Western blotting. HeLa cells were transfected with the indicated plasmid constructs and subjected to immunoprecipitation with anti-TRIM28 antibodies. Input material, i.e., aliquots of extracts (labeled “X”; about 1/40th of extract submitted to immunoprecipitation) was loaded side by side with the respective eluate from Protein G beads (“IP”). The top panel (**a**) interrogated the successful pulldown of the bait TRIM28 (anti-TRIM28 antibody staining; black arrow) whereas the bottom panel (**b**) stained for respective co-immunoprecipitated GST-KRAB fusion proteins (anti-GST; open arrow). The asterisk marks proteins reactive with secondary antibodies that coeluated from beads (note the lanes from the mock transfection). They likely represent Protein G and IgG light chain (from the immunoprecipitating antibody) protein species.

Next, we evaluated whether transcriptional repressor activity of KRAB mutants coincided with their ability to form complexes with TRIM28. We transfected plasmids encoding selected KRAB domains as fusion proteins with a glutathione S-transferase (GST) tag and evaluated whether they associated with endogenous TRIM28 and therefore could be pulled-down with anti-TRIM28 antibodies (Figure 5). The GST-ZNF10-AB fusion protein was very efficiently co-immunoprecipitated with TRIM28. The negative control, a ZNF10-AB mutant with double proline insertion before the glutamate in the VMLE motif (ZNF10-PP-AB), was not pulled-down at all, as expected [33]. Similarly, the PRDM9 aKRAB-A fusions extended with authentic residues up to position 97 (P9A/24-97) or with ZNF10-B both failed to form complexes with the cell’s TRIM28. The analysis of PRDM9-KRAB-A mutants attached to ZNF10-B revealed that P9Am10-Z10B as well as P9Am11-Z10B was co-immunoprecipitated with TRIM28. This indicated the existence of stable complexes between these aKRAB-A mutants and TRIM28. In contrast, P9Am12-Z10B and P9Am13-Z10B proteins were not pulled down with TRIM28 at all. Based on the blot intensities of the pulled-down proteins, the extent of complex formation between transcriptionally active heterologous KRAB fusions and TRIM28 appeared to be reduced compared to ZNF10-AB. However, there were also differences in expression of the plasmid-encoded GST-fusions in the input extracts, and immunoprecipitation is not a very quantitative methodology.

Altogether, the data demonstrated that the KRAB domain property “complex formation with TRIM28” is strongly positively correlated with the function “transcriptional repressor activity in heterologous reporter assays”: The PRDM9 mutants P9Am10-Z10B and P9Am11-Z10B that displayed robust transcriptional repression stably interacted with TRIM28, while mutants P9Am12-Z10B and P9Am12-Z10B, inactive in the repression assay, did not.

### 2.5. Comparative Topologies of Ancestral versus Modern KRAB Domains

**Figure 6:**
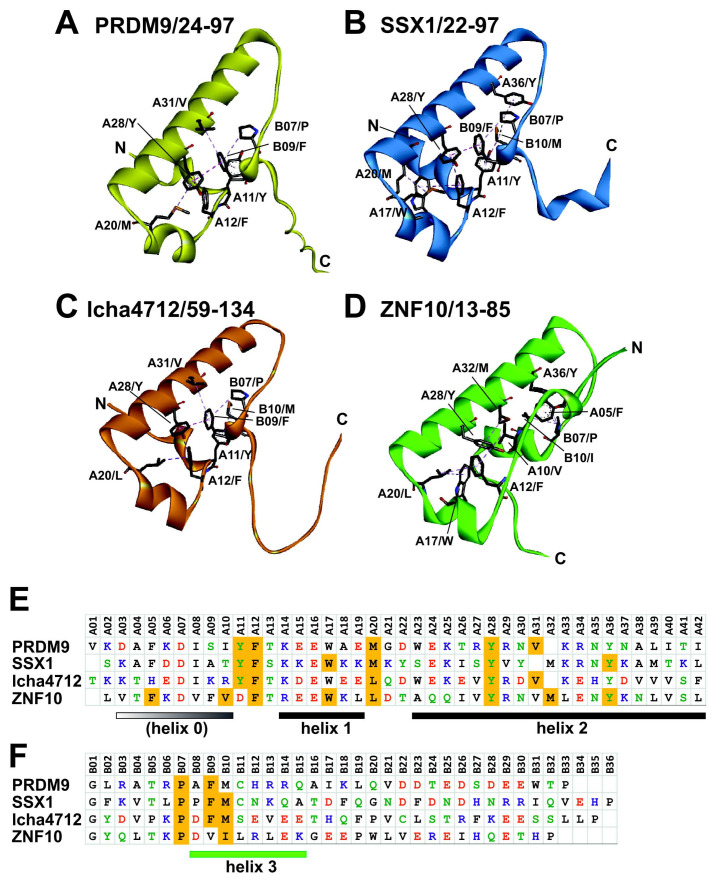
Structural models of ancestral and modern KRAB domains with focus on the global arrangement of characteristic α-helices. Models computed by AlphaFold2 for human PRDM9 aKRAB (NP_064612; **A**), human SSX1 aKRAB (ENSP00000366118; **B**), coelacanth KZNF lcha4712 aKRAB (ENSLACP00000004712; **C**) and human ZNF10 mKRAB (NP_056209; **D**). The amino acid residues used as input for modelling are indicated after the protein names. They refer to the positions in the whole protein. The protein termini of the models are labeled by the letters N and C. The cartoon representation of each protein’s main chain is color coded for PRDM9 (yellow), SSX (blue), lcha KZNF (orange) and ZNF10 (green). Alignments for KRAB-A (**E**) and KRAB-B (**F**) are shown on the bottom with the residues detailed in the structures highlighted by orange shading. This set of amino acids form hydrophobic interactions (broken lines) within the body of a KRAB and are interpreted to participate in forming the characteristic topology. The characteristic helices are named below the alignments. Helix 1 and 2 (black bars) are found in ancestral and modern KRAB domains. A stable Helix 3 (green bar) in KRAB-B is typical for modern KRAB. Helix 0 (graded white to black shading) is more or less pronounced depending on protein.

To identify possible structure-function relationships that might explain our experimental results, we analyzed 3D models of KRAB domains using the Colab-Fold/AlphaFold2 protein prediction resource [59,60]. All structures that describe and discuss different structural aspects can be found as PDB 3D files in the Supplement PDB.zip. Overall, ancestral and modern KRAB domains displayed a high level of shared topology built of usually three core α-helices of which the most extended, helix 2, seems to be completely conserved (Figure 6). Even the evolutionary oldest member of PRDM9 from mussel conforms to this 3-D structure (see below). The core structure usually consists of a short helix 1 (A14-A19) that turns by an almost 90-degree angle in the same plane to the central helix 2 that starts at A23 to proceed until the end of KRAB-A. The consecutive sequence of the KRAB-B subdomain is more flexible but between B08 and B15 also shows some helical structure. However, the propensity is rather weak for the aKRAB-B members PRDM9, SSX1 and lcha4712. In contrast, the mKRAB-B of ZNF10 displays a specific well-built helix, that we named helix 3. Importantly, this helix 3 crosses behind helix 2 in an almost orthogonal manner but skewed in space. The KRAB core structure appears to be mostly mediated by hydrophobic interactions of amino acids that are conserved in the respective KRAB subgroups: Examples are A12/F, A20/M or L, A28/Y and B07/P for all KRAB subtypes, A11/Y and B09-10/FM that are specific for the ancestral subtypes or A10/V and B10/I for modern KRAB. The spatial arrangement of mKRAB-B of ZNF10 seems to be supported by electrostatic interactions between B06/K and B14E as well as the peptide bond oxygen of B13/L with A29/R. Note that some KRAB molecules have propensities of further α-helices, such as SSX1 at the KRAB-A N-terminus (“helix 0”) or at positions beyond residues assigned to helix 3 in ZNF10-B.

**Figure 7:**
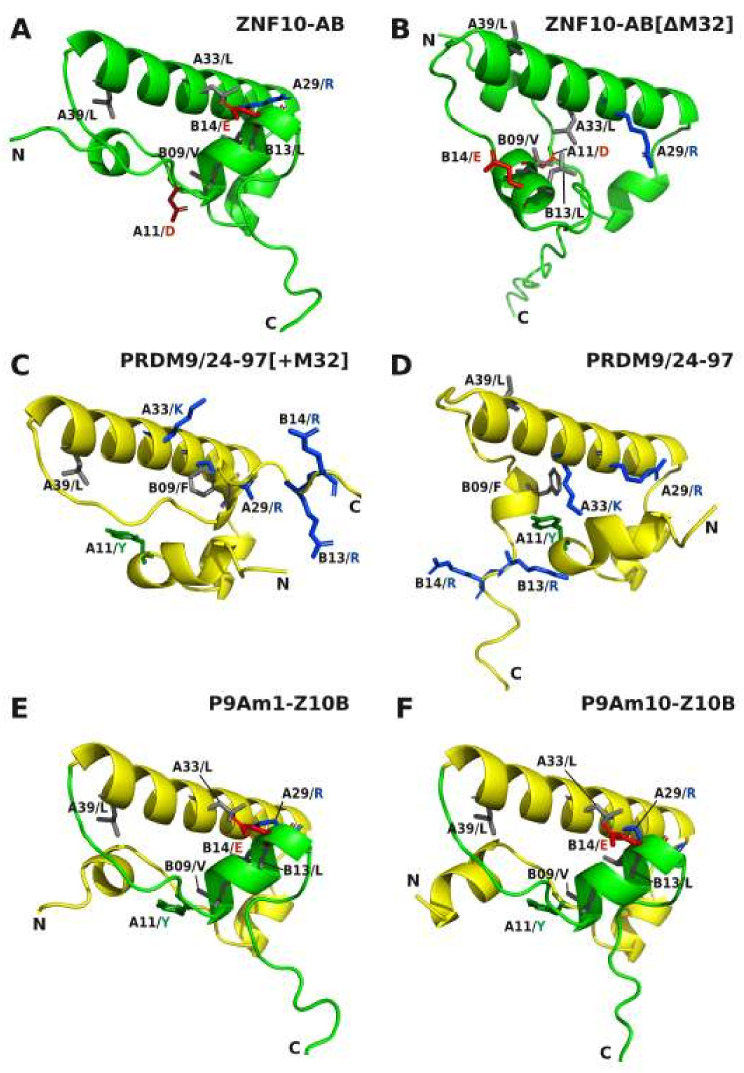
Structural models of selected KRAB domains to highlight important changes in helical and side-chain orientations in mutants and hybrids with ZNF10-B compared to wild-type modern ZNF10-AB. Depicted are views of structures computed with AlphaFold2 for the following KRAB domains: Wild-type ZNF10-AB modern KRAB (**A**), a ZNF10-AB mutant that lacks the A32/M residue (**B**), the human PRDM9 aKRAB with an inserted residue A32/M (**C**) or in wild-type configuration missing this residue (**D**), the human PRDM9 KRAB mutant with A32-34/MLE as hybrid with ZNF10-B (**E**) and the PRDM9 mutant aKRAB-A hybrid with ZNF10-B with the minimal number of amino acid changes compared to wild-type PRDM9 aKRAB-A that showed full repression potential (**F**). Highlighted amino acids side-chains reflect either differences between mKRAB of ZNF10-AB and aKRAB or PRDM9 (e.g., A11/D vs. A11/Y; A33/L vs A33/K; B09/V vs B09/F; B13-14/LE vs. B13-14/RR) or are used as positional markers for orientation (e.g., A29/R; A39L).

As described above, the ancestral aKRAB-A sequences are all characterized by being one amino acid short at position A32 that usually is a conserved M in functional mKRAB-A domains. Indeed, a necessary, though not sufficient, step was to insert MLE at A/32-34 that provided the additional M for any PRDM9 mutant to be functionally active. AlphaFold2 models indicated that insertion of A32/M rearranged the 3-helix core structure with respect to the spatial relationship between core helix 2 and the KRAB-B helix 3. This is obvious in the model of ZNF10-AB wild-type (with A32/M) compared to the one with deleted methionine (Figure 7A vs. Figure 7B) as well as in the comparison of the models of wild-type PRDM9/24-97 versus its counterpart with A32/M insertion (Figure 7D vs. Figure 7C). At the same time, the side-chains of amino acids following the A32 position are oriented differently depending on presence/absence of A32/M. They are turned by about 97 degrees. This is illustrated when looking at A39/L in comparison to A29/R, which we use here as positional marker (Figure 7). The structures of mutants P9Am1-Z10B and P9Am10-Z10B look very similar, especially the positioning of the core helices with the specific spatial relationship of helix 3 to helix 2. However, only mutant 10 was fully active and stably interacted with TRIM28. Thus, while the overall structural topology is necessary, the side-chains, i.e., their physicochemical properties, must fit to obtain full functionality.

In conclusion, the transition from ancestral to modern KRAB required: (i) the insertion of a permissive amino acid like methionine at position A32 to confer the necessary spatial change of aKRAB-A into a core structure of three helices with correct spatial relationships; (ii) attachment of mKRAB-B of ZNF10 to deliver an appropriate helix 3, and (iii) specific side-chain changes that likely reflect a specific binding surface for interacting proteins, in particular for TRIM28.

## 3. Discussion

The advent of AlphaFold2, the AI system developed by DeepMind [59], now empowers detailed in-silico modelling of 3-D structures of any human protein. While recent work has made progress in postulating a KRAB-binding protein interface within the coiled-coil domain of TRIM28 [61,62], information on KRAB structure itself is scarce. Prior to AlphaFold2 implementation, there was just one experimentally determined KRAB structure in the PDB structural protein databank, model 1V65. It represents an otherwise undescribed NMR structure of a mouse protein (RIKEN 2610044O15) with mKRAB-A linked to a weak KRAB-C protein. This structure supports the core helices 1 and 2 computed by AlphaFold2 for our KRAB domains under investigation. Further, an AlphaFold2 model of this mouse protein (AlphaFold Protein Structure Database at https://www.alphafold.ebi.ac.uk/; accessed on 2 November 2021) matches the NMR-derived structure well. Results from biophysical investigations of recombinant proteins pointed to the possibility that a canonical KRAB domain displays a non-rigid malleable structure that adopts a more stable 3-D fold upon binding to TRIM28 [34,63]. Experiments with amino acid exchange mutants pinpoint essential features of KRAB-A for its functions “recruitment of TRIM28” and “transcriptional repression” ([50,51,53,64]; this paper). However, until now, we have lacked a sound theoretical framework that can be used to stratify the multitude of existing KRAB domains in mammalian genomes with respect to binding strength to TRIM28 and transcriptional repressor potency. Among the unresolved questions are the roles of the different KRAB-B subdomain configurations for function. While a B subdomain has been shown to be essential for repression and TRIM28 interaction ([33,34,35]; this paper), it is not yet possible to predict from an mKRAB-A peptide sequence whether it needs a B-type subdomain for function.

**Figure 8:**
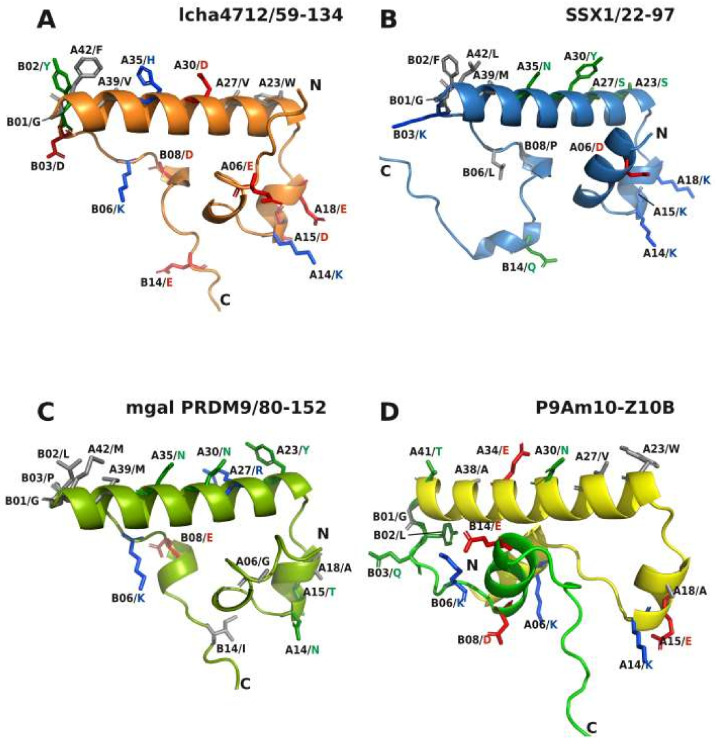
Structural models highlighting amino acids that are proposed to define potential binding interfaces for proteins interacting with KRAB domains. AlphaFold2 models of coelacanth aKRAB of KZNF lcha4712 (**A**), human SSX1 (**B**), mussel (mgal = *Mytilus galloprovincialis*) PRDM9 (**C**) and of P9Am10-Z10B, the mutant PRDM9 aKRAB-A hybrid with ZNF10-B that behaves as a *bona fide* mKRAB domain (**D**). The residues with depicted side-chains reflect the positions described in Figure 9 with focus on amino acids whose side-chains point outwards from the main body formed by helices 1 to 3. Note that the spatial orientation of helix 1 towards helix 2 is very similar for aKRAB (**A**–**C**) as well as mKRAB (**D**) as exemplified by the depiction of side-chains of A15 and A18 versus A30. In contrast, helix 3, represented e.g., by B14, has a completely different spatial layout in aKRAB compared to mKRAB. Thus, the mKRAB hybrid presents a 3D structure highly similar to the 3-D structure of ZNF10 in Figure 6D.

**Figure 9:**
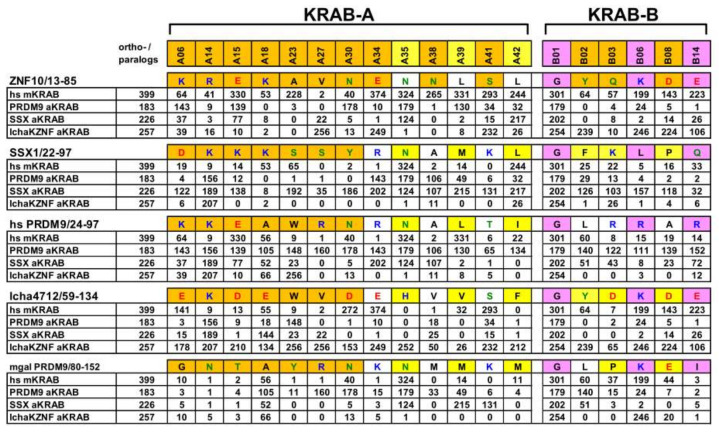
List and frequency distributions of amino acid residues at positions that are considered to be of structural and functional relevance for the KRAB domain in its ancestral or modern configuration. Position-specific counts suggest the existence of a specific aKRAB and mKRAB protein-protein interaction code with other proteins or with components that are not part of the KRAB domain. Five sequences were chosen as blueprints for each specific group. They include human ZNF10 KRAB-AB (residues 13–85) as a mKRAB-AB, human SSX1/22-97 for the SSX aKRAB subgroup, human PRDM9(24-97) to represent the PRDM9 aKRAB subgroup and KZNF lcha4712/59-134 for the coelacanth aKRAB group. Appended is the mussel (mgal = *Mytilus galloprovincialis*) PRDM9/80-152 (Uniprot A0A3L5TRV6) as the aKRAB with the deepest evolutionary root. The interrogated positions were chosen based on the respective AlphaFold2 models (depicted in Figure 7 and Figure 8; original PDB files can be found in the supplements). They represent residues whose side-chains point outward from the main body of KRAB in general (orange shading) or of aKRAB-A specifically (yellow shading). In the latter case, because of the missing A32 position compared to mKRAB-A, these residues likely reflect interacting AA of aKRAB-A domains. Residues assumed to be structurally relevant for shaping mKRAB-B as in the blueprint of ZNF10-B are depicted in pink shading. The distribution of each shown residue of a blueprint was scored against the groups of human mKRAB, the PRDM9, SSX and coelacanth KZNF aKRAB sequences by counting their frequencies. Note that counting of amino acids in sequences following KRAB-A was done irrespectively of the existence of an actual conserved KRAB-B. A more extended table considering all position of KRAB can be found in Table S2.

In general, our comparative 3-D structural analyses made with AlphaFold2 revealed the following characteristics of the KRAB domain. The core 3-D topology built from 41–42 residues of KRAB-A together with subsequent sequence of up to 35 amino acids of KRAB-B is mainly made of α-helices that are linked by unstructured stretches of amino acids (Figure 6). The amphipathic shorter helix 1 and longer helix 2 appear to form a conserved stable L-shaped conformation. It was found in all KRAB-A structural models and does not per se distinguish ancestral and modern KRAB domains. This common 3 D structure already existed more than 700 million years ago before the split of *Bilateria* in *Protostomia* and *Deuterostomia* as evinced by its presence in mussel PRDM9. However, helix 3 is only poorly formed in aKRAB-B models (Figure 7D) while the modern KRAB helix 3 exemplified by mKRAB-B of ZNF10 is very visible and specifically arranged perpendicular to helix 2 in a skewed position (Figure 7A and Figure 8A–C). This typical spatial arrangement of helix 3 in the modern KRAB domains is dependent on interactions with amino acids in helix 2. In case of ZNF10, the participating residues are B13-14/LE and A29/R (Figure 7A). Importantly, this specific topology composed of helix 3 of mKRAB-B and central helix 2 determines the spatial orientation of amino acid side-chains pointing outwards in helix 2. Note, that the introduction of an additional amino acid in helix 2 (A32/M), the most characteristic difference between ancestral and modern KRAB-A (Figure 1), turns the side-chains by 97 degrees with respect to each other. This is easily visible when looking at A29/R and A39/L (Figure 7A,B). Thus, this insertion completely changes the nature of outward-pointing residues of the helix 2 surface of mKRAB-A providing a different physicochemical landscape for intra or intermolecular interactions (Figure 8 and Figure 9, extended version in Table S2). It is tempting to speculate about the existence of a KRAB specific amino acid interaction surface code that is correlated to graded binding of the hundreds of different KRAB domains to TRIM28 in an ordered mode.

Our structural analysis with AlphaFold2 focused on the KRAB domain. Consequently, we input only the amino acid sequence representing this domain into AlphaFold2. Our KRAB models were reproducibly predicted with overall high confidence. However, we would like to emphasize that the AlphaFold2 algorithms only provide the most likely snapshot of a 3-D structure. This snapshot represents a particular protein conformation that may be of functional relevance but that does not necessarily cover protein dynamics or protein states that KRAB adopts upon interaction with molecules such as TRIM28 or after post-translational modifications. The confidence scores given by Alphafold2 for our KRAB 3D models were overall high (>70) or even very high (>90) for the central domain region containing the characteristic alpha-helical conformations (predicted per-residue local-distance difference test value pLDDT; [59]; see example ZNF10-AB sequence and scores in Figure S2A,B). High confidence prediction for a protein’s backbone was observed to correlate with high side-chain accuracies [59]. Therefore, we judge the positional landscape of specific side-chains that we highlighted for function to be of significance. Only the unstructured tails, in particular at the C-terminal end of KRAB-B, were scored low (pLDDT < 70) or even very low (pLDDT < 50). 

The ZNF10 full-length protein, as included in the AlphaFold database (https://www.alphafold.ebi.ac.uk/entry/P21506 (accessed on 10 January 2022); confidence scores and snapshot shown in Figure S2C,D), illustrates the complexity of the 3D protein topology of this KZNF protein. While the KRAB domain and the typical C2H2 zinc finger folds (each made of a two-stranded antiparallel β-sheet and an α-helix) are structurally well defined, the whole protein model contains extended regions of intrinsic disorder. The largest unstructured regions are predicted to exist in the spacer part between KRAB-AB and the first zinc finger. This may reflect a necessary spatial arrangement that allows the zinc fingers to bind their cognate DNA motif with high degrees of freedom while leaving the KRAB domain flexible to engage the TRIM28 hub protein with all its associated proteins. In addition, these regions may permit transitions in conformational states often found for proteins with intrinsically disordered domains and typical for transcription factors [65].

Our experimental data show that the ancestral KRAB-A domain of human PRDM9, which belongs to the group of KRAB domain-encoding genes with the deepest roots in evolutionary time, requires only three distinctive changes of amino acid residues at specific positions to transform it into a canonical modern KRAB-A. These changes consist of the placement of three hydrophobic side-chain residues (F9, L20, V27) and an essential MLE insertion (residues 32–34). The most prevalent amino acids at positions A20 and A27 in the human mKRAB-A logo (Figure 1E) are indeed hydrophobic amino acids, in both cases leucines. The MLE insertion replaces a two-amino acid KR positively charged piece in PRDM9 and resulted in a proposed 3D model that displays the characteristic three-helix core structure of mKRAB if connected to a proper helix 3 provided by the mKRAB-B domain of ZNF10. The parallel gain in transcriptional repression activity and complex formation with TRIM28 suggests that the highlighted amino acids have properties that are likely necessary, or at least favorable, to contribute to the molecular interface between KRAB-A and TRIM28. This is supported by structural evidence in models of the P9Am1-Z10B versus the P9Am10-Z10B mutant (Figure 7). Both present an identical 3-D structure, but only the latter one with the specific residue replacements showed complete repressor activity. Thus, in addition to the correct topology, a specific physicochemical landscape is required to confer function. This landscape is probably mainly determined by freely available side-chains of amino acids pointing outward from the core KRAB body to enhance or mitigate interactions with other proteins, in particular with TRIM28. The specific orientation of such potential binding interfaces of the different aKRAB subtypes and mKRAB are illustrated in Figure 8, and frequency counts of the involved amino acids are shown in Figure 9. One of the best examples concerns A34/E in mKRAB domains, which are highly conserved (see Figure 1E) and part of the MLE motif necessary for potent repressor activity of ZNF10 and mutant P9Am10-Z10B ([51], this paper). The essential role of A34/E for KRAB function has been emphasized by the huge gain of repressor potency and protein interaction with TRIM28 when the cognate A34/G residue of ZNF746 KRAB-AB was replaced by E [50]. 

Recent work presented a mutational deep scan of the ZNF10 KRAB-AB domain using a novel high throughput approach with a lentivirally transduced library of KRAB mutants fused to a TetR DNA binding domain and next generation sequencing readout [66]. Using this approach, the authors defined residues in ZNF10 KRAB-AB that are important for potent transcriptional silencing (see Table S2, lower part). At position A32/M, replacement with nine other amino acids was strongly detrimental and, at position A34/E, 11 substitutions had considerable negative effects on transcriptional repression. Thus, this experimental study highlighted the importance of A32-34/MLE motif in mediating repressor activity. Note that proline, as a known helix-breaker, abolished repression activity when AlphaFold 2 predicted α-helical structures. (see [66]; Table S2). In addition, a strong negative effect was seen as well upon substituting A16/E for P in helix 1. However, one has to keep in mind that amino acids always function in concert with other residues in the context of a protein. Thus, in the case of substitutions such as A20/M to L and A27/R to V, single amino acid scanning effects in ZNF10 might not coincide with experimental data and substitutions conferring repressor activity to PRDM9 aKRAB/mKRAB-B hybrid proteins. Apparently, amino acid replacements can have the greatest effects if structural features are altered that might result in the dislocation of more than one AA side-chain. Thus, functional readouts of single amino acid substitutions may be dependent on the overall configuration and neighboring amino acids if they differ from PRDM9 to ZNF10-KRAB-A. In the case of R instead of A27/V, the deleterious effects might be many-fold. Maybe, arginine either interferes with TRIM28 binding due to its bulkiness and/or its charge. Based on the frequency distributions, hydrophobic amino acids are preferred at that position and the presence of R is rare (see Table S1). 

Previous knowledge and our compilation of aKRAB sequences suggest the following scenario for the evolution of KRAB. The likely progenitor of the modern KRAB domain, aKRAB-A of PRDM9, can be traced back to members of the clade *Protostomia* and is present in the genome of most vertebrate branches including primates, but not in birds, crocodiles and amphibians ([14]; this paper Table S1). Since the N-terminal domain configuration of aKRAB-A and SSXRD domains is shared by the SSX group of proteins, the respective genes likely derived from PRDM9 ancestors. SSX orthologs appear to be confined to mammals ([47]; this study Table S1). This observation argues that the split from PRDM9 took place in the last common ancestor of mammals. PRDM9 as well as SSX usually have only a few paralogs within the same species, e.g., the human genome harbors two PRDM9 (PRDM9, PRDM7) and 11 SSX (SSX1, SSX2, SSX2B, SSX3, SSX4, SSX4B, SSX5, SSX6P, SSX7, SSX8, SSX9) proven or potential protein-coding paralogs (see Table S1). In both aKRAB-encoding gene groups the different domains are encoded on different exons. Interestingly, both gene groups are predominantly expressed in testis.

The third group of genes encoding an aKRAB-A has been found so far only in the coelacanth *Latimeria*, a genus of sarcopterygian (lobe-finned) fish. This group (named lcha KZNF in Figure 1D) already has the domain configuration such as the modern KZNF proteins in that a KRAB domain, here containing aKRAB-A, and is teamed up with C2H2 zinc finger modules, but with neither SSXRD nor Pr/SET. The gene class multiplied to more than 200 paralogs in the coelacanth genome ([11]; this study). However, unlike PRDM9, SSX and modern KZNF genes, lcha KZNF genes usually encode the respective protein only on a single exon [11]. Importantly, the coelacanth contains a TRIM28 ortholog that interacts with coelacanth aKRAB-ZNF proteins, but not with a modern-type KZNF protein [48]. Thus, the common ancestor of the coelacanth and tetrapods had everything in place for ongoing evolution of the KRAB/TRIM28 system to the current situation in land vertebrates. It will be interesting to determine whether the lungfish already contains changes that bring the KRAB/TRIM28 system closer to the tetrapod/mammalian situation. The lungfish was postulated to be the closest extant fish relative to tetrapods rather than the coelacanth [67]. Its genome harbors not only a TRIM28 ortholog [33] but appears to contain an expanded set of KRAB domain encoding genes [68].

Our sequence compilation enabled us not only to distinguish ancestral and modern KRAB-A, but to also define motifs in the sequences subsequent to KRAB-A that can be considered ancestral KRAB-B or KRAB-B precursors. The closer phylogentic relationships of aKRAB from coelacanth KZNFs to modern KRAB is also visible in KRAB-B: Highly prevalent residues B02/Y, B06/K and B08/D of the coelacanth aKRAB-B sequences are typical for the mKRAB-B of human KRAB domains but are not found in PRDM9 and SSX ortholog/paralog groups (see logos in Figure 1 to the right; frequency distributions in Table S2 upper part). Even the dominant B14/E of mKRAB-B in human sequences is present in a considerable number of lcha KZNF aKRAB-B (106/257) compared to low frequencies in the SSX group (26/226) and almost absence in PRDM9 orthologs (1 out of 183, see frequency counts in Figure 9). In summary, an ancestral primordial KRAB B domain becomes apparent that shares common amino acids with human KRAB-B sequences, plus presenting the FM motif at B09/B10 which is not found in human KRAB-ZNF proteins but shared by the aKRAB subgroups.

With the support of AlphaFold2, informative AA positions common to aKRAB and mKRAB domains have been postulated in helix2 and partly in helix1 and helix3 to define an AA interaction code to enable and to mediate graded protein-protein interactions (Figure 9). Note that PRDM9’s aKRAB-A is involved in complex formation to proteins functionally related to meiotic recombination [43,69]. The guanine nucleotide exchange factors RAB3IP and SSX2IP have been described as proteins that interact with the N-terminal region of SSX2 containing the aKRAB domain [70], but their functional impact remains unclear as do most of the cellular roles of SSX proteins. It is interesting to note that the SSX1/2 part of the fusion proteins with SS18 derived from the t(X;18) translocation in synovial sarcoma does not contain the aKRAB of SSX [39,47]. This fact might have functional consequences for tumor biology. For mKRAB domains, the most important interaction partner is TRIM28 and most human KZNF proteins with mKRAB domains indeed interact with TRIM28. Yet, specific sets of distinct proteins were found in complexes with tagged, exogenously expressed KZNF proteins [48,71]. However, it remains to be determined which interaction is direct without a bridging molecule, and whether the mKRAB domain is involved in the interaction interface. The KRAB-O protein provides an example for direct binding because it not only binds with its canonical mKRAB to TRIM28 but at the same time also to the Sry (sex-determining region Y) protein [52]. Our data here and in previous work on ZNF746-AB [50], highlight positions A27 and A34 as residues that should directly interact with TRIM28 (Figure 8, Table S2).

## 4. Methods

### 4.1. Compilation and Computational Analysis of Sequences

Gathering of “ancestral” KRAB-A amino acid sequences (abbreviated aKRAB-A) started from the entries contained in InterPro IPR003655 (www.ebi.ac.uk/interpro/; release 77.0; 14 November 2019) under the header “Krüppel-associated box-related”. Entries are listed based on UniProt accession numbers. They are distinguished in the InterPro database from bona fide Krüppel-associated box (KRAB) sequences (IPR001909). Additional information was added to each entry using UniProt batch search based on the UniProt identifiers. This included, most importantly, protein sequence and species allocation as well as cross-database identifiers and annotations such as e.g., ENSEMBL IDs, genomic location, ENTREZ gene IDs and PFAM domain hits. Curation was done in several steps and included iterative use of HMMER software (HMMER v2.3.2 software obtained from www.hmmer.org (accessed on 9 February 2011) and installed locally) as well as individual TBLASTN searches with different PRDM9 orthologs against ENSEMBL and NCBI genome and transcriptome databases for selected species. Initially, a CLUSTALW alignment (www.genome.jp/tools-bin/clustalw; accessed from 25 March 2020 to 8 December 2021) of manually compiled aKRAB-A sequences from PRDM9 and SSX1 orthologs (130 sequences) was used to make a profile hidden Markov model (HMM) for this domain with “hmmbuild” of HMMER v2.3.2. This initial aKRAB-A HMM was used to extract the aKRAB-A domain sequences and remove all sequences in the InterPro data that had E-values ≥ 0.01 when using hmmpfam of the HMMER software. The resulting 491 entry aKRAB-A sequences were used to build an updated profile HMM. Next, the entries were consolidated within each species by removing obvious duplicates that derived from different gene models of the same genomic locus or protein isoforms. Further, the updated profile HMM was applied with a cutoff of E-value 1E-10 to remove sequences with large truncations or incomplete sequence information and spurious results that are due to stochastic sequence similarities. Additionally, we did cross-checks with studies that include KRAB-A-related sequence lists (PRDM7/9 orthologs: [14]; aKRAB-A sequences in *Latimeria chalumnae*: [11]). Sequences not contained in the InterPro-derived list were then manually appended. Similarly, we added the PRDM9 ortholog sequence we identified by reciprocal BLAST searches of human PRDM9 and *Latimeria chalumnae* PRDM9 in the transcriptome of lungfish Protopterus annectens [72]. A final overall profile HMM was built by aligning the aKRAB-A sequences extracted from the final list of 664 entries and using the hmmbuild tool of HMMER (v2.3.2). Finally, the total list was divided into subgroups based on orthology, domain composition and annotations. These subgroups were PRDM9 orthologs, SSX orthologs and aKRAB-A-ZNF proteins from *Latimeria chalumnae*. We built subgroup-specific aKRAB-A profile HMMs that are provided as HMMs.zip. The human KRAB-A sequences (416 entries after manual removal of human PRDM9, PRDM7 and frog XFIN sequences) were taken from [36], their Table S2. 

The statistical significance of differences between the E-value distributions of the different subgroups was tested after –log10 transformation of the values with a two-sided Wilcoxon-Mann-Whitney test (Ian E. Holliday 2017, Wilcoxon-Mann-Whitney Test (v1.0.6) in Free Statistics Software (v1.2.1), Office for Research Development and Education, URL https://www.wessa.net/rwasp_Reddy-Moores%20Wilcoxon%20Mann-Witney%20Test.wasp/; accessed on 25 June 2020). Entries that were scored below the E-value threshold of 0.01 for a particular profile HMM using hmmpfam (empty E-value cells in Table S1) were set to this threshold which resulted in a –log10 value of 2 for the calculation of the median and the statistical tests.

### 4.2. Sequence Logos and Tree Construction

The computed ClustalW alignments were input into the webtool Skylign [73]; http://skylign.org/; accessed from 25 March to 11 December 2020) with the options “Create HMM—remove mostly-empty columns”, “Some sequences are fragments” and “information content—above background”. Since KRAB domains usually have a well-defined size without many gaps or insertions there was no added value to display the parameters “occupancy”, “insert probability” and “expected insert length”. The numbers have, therefore, not been displayed. Logos were exported from the website as svg vector files, the color scheme of the amino acids was changed by a text script and the logos were labeled within CorelDraw X8 for the final figures. The telltale gap between position 31 and 32 of aKRAB-A sequences was introduced manually for highlighting.

Selected KRAB domains aligned with ClustalW were used to infer a phylogenetic tree by the Maximum Likelihood method and a JTT matrix-based model within the software package MEGA X v 10.1.8 [74]. The bootstrap consensus tree was derived from 1000 replicates. Branches based on partitions in less than 50% of the replicates were collapsed and the final tree horizontally condensed for the final figure. The species tree was built with selected species as input and the “Build a TimeTree” function at http://www.timetree.org/ (accessed on 25 August 2021) [49]. 

### 4.3. Protein 3-D Structure Models

Structural models for selected KRAB domains were computed using AlphaFold2 [59] using ColabFold at https://colab.research.google.com/github/sokrypton/ColabFold/blob/main/AlphaFold2.ipynb (accessed between 31 October and 13 December 2021) [60]. For each KRAB sequence, we usually obtained three models from the server and worked with the rank1 model that represents the computation with best score. The obtained PDB files were inspected and processed for figure making using PyMOL™ Molecular Graphics System, Version 2.5.0 (Schrödinger, LLC, New York, NY, USA) and Discovery Studio Visualizer v4.5.0.15071 (Biovia Corp.). Specific 3D views were rendered and exported as PNG files and imported into CorelDraw for labeling.

### 4.4. Cell Culture and Transfection

HeLa cells were originally obtained from the German Cancer Research Center (Heidelberg, Germany) while the HAP1 wild-type (C631), TRIM28 knockout cell line clone T28KO1 (HZGHC000293c001) and the HAP1 SETDB1 knockout cell line SETDB1KO (HZGHC001331c001) were purchased from Horizon Genomics (Vienna, Switzerland). Cells tested negative for mycoplasma infection using a commercial assay (LT07-418 Lonza, Cologne, Germany). Cells were grown at 37 °C and 5% CO_2_ in DMEM (HeLa) or IMDM (HAP1) supplemented with 10% fetal calf serum (heat-inactivated, FBS Superior S0615, Merck/Sigma-Alrich GmbH, Taufkirchen, Germany) and 50 units/mL penicillin and streptomycin, each. HeLa cells were transfected with FugeneHD (E2311, Promega GmbH, Walldorf, Germany), whereas HAP1 cell lines were transfected with Turbofectin 8.0 (TF81001, OriGene Technologies GmbH, Herford, Germany) according to the manufacturer’s recommendations at 3 µL transfection reagent per 1 µg of DNA.

### 4.5. Immunoprecipitation and Western Blotting

Protein extract generation, immunoprecipitation and Western blotting methods were essentially performed as previously described [33]. Briefly, cells grown in 10 cm-dishes were harvested by scraping them in PBS and then lysed in buffer TST (20 mM TRIS/HCl Ph 7.5; 60 mM KCl, 15 mM NaCl, 10 mM MgCl_2_, 1 mM CaCl_2_, 250 mM Sucrose, 0.5% Triton X-100), freshly supplemented with 1 mM DTT, CompleteUltra EDTA-free protease inhibitors (Roche, Merck/Sigma Aldrich GmbH, Taufkirchen, Germany) and 1 mM sodium orthovanadate, 40 mM beta-glycerophosphate phosphatase inhibitors. Immunoprecipitation was done with mouse monoclonal antibodies against human TRIM28 (mAb 1Tb1A9, a generous gift of Dr. Pierre Chambon; [75]; 5 µg antibody/sample). Complexes were pulled down with magnetic protein G beads (GE Healthcare 28-9440-08) and enriched proteins eluted with 1 × Lämmli SDS sample buffer. Standard 12% Lämmli-type SDS polyacrylamide gels were loaded with the extracts and submitted to semi-dry electroblotting. Blots were stained with Ponceau S red and cut into an upper part (molecular weight region top to above 55 kD) and a lower region (below 55 kD to bottom). The middle region around 55 kD was omitted since it contained the immunoglobulin heavy chain from the immunoprecipitating antibody. Strong staining of this immunoglobulin chain with secondary antibodies can obscure weaker signals on the blot. TRIM28 protein was detected by a mouse monoclonal anti-mouse TRIM28 antibody (cross-reactive to human TRIM28, BD Biosciences #610680) while staining of GST fusion proteins was done with goat anti *Schistosoma mansoni* glutathione S transferase antibody (at 0.19 µg/mL; Rockland 600-101-200, Biomol GmbH, Hamburg, Germany). We used the secondary fluorescent dye-conjugated antibodies goat anti-mouse IgG-IRDye680 (#926-32220, LI-COR Biosciences GmbH, Bad Homburg, Germany) and donkey anti-goat IgG-IRDye800CW (#926-32214, LI-COR Biosciences GmbH, Bad Homburg, Germany). Signals were recorded with a Li-Cor Odyssey CLx imager (LI-COR Biosciences GmbH, Bad Homburg, Germany). Color or greyscale images were exported in PNG format and subjected to the command “auto contrast” within Adobe Photoshop CS4.

### 4.6. Luciferase Reporter Assays

We used our previously described dual luciferase system [33,50]. It is based on a firefly (*Photinus pyralis*) luciferase gene under control of a strong SV40 promoter with five upstream binding sites for the DNA-binding domain of yeast transcription factor Gal4 (Gal4-DBD). For normalization we used a plasmid expressing *Renilla* luciferase as reference reporter. For each well of a six-well plate we used here 500 ng firefly luciferase construct pGL3-5′Gal4BS_5_, 10 ng *Renilla* vector pGL4.74 (hRluc/TK, Promega GmbH, Walldorf, Germany) and 1500 ng effector construct based on pM3. Enzyme activities were measured with the dual luciferase assay system (Promega GmbH, Walldorf, Germany) 24 h after transfection. The effectors, i.e., the KRAB domains were expressed as fusion proteins with Gal4-DBD. Their expression was verified by Western blotting (Figure S1). Results for the Gal4-DBD alone were used as baseline and set to one. Repression was calculated by dividing the normalized firefly luciferase activity of the Gal4-DBD baseline by the activity of the KRAB domain fusion to be investigated within each experiment. Box plots were made by inputting the data into the R computing environment accessed through the dedicated web service http://shiny.chemgrid.org/boxplotr/ (accessed 8 July 2020). Center lines depict the median values, the box borders indicate the 25th and 75th percentiles and the whiskers are plotted according to Tukey.

### 4.7. DNA Constructs

Constructs that encode ZNF10-KRAB-AB, the mutant ZNF10-KRAB-PP-AB, wild-type PRDM9-A and PRDM9-A/24-97 have been described before [33]. DNA fragments with flanking restriction sites (XhoI/SalI) that encode the other desired KRAB sequences (mutants) were obtained by commercial gene synthesis (Eurogentec, Seraing, Belgium). They were then cloned into eukaryotic expression vectors pM3 and pN2-GST [33] using the vectors’ SalI sites. The pM3 effector constructs encode an N-terminal Gal4 DNA binding domain (Gal4-DBD) attached to the investigated KRAB domain for use in reporter assays. The pN2-GST vectors attach an N-terminal glutathione S-transferase (from *Schistosoma japonicum*) portion to the desired C-terminal KRAB part as a tag for protein-protein interaction studies. 

## 5. Conclusions 

Our structure-function analysis in concert with AlphaFold2 3D modeling postulates structural and functional principles for a better conformational understanding and causal stratification of the KRAB transcriptional repressor domain. We describe amino acid landscapes that distinguish modern and ancestral members of the family. Our HMM models for the different subfamilies should prove useful to improve domain designations in scientific databases. It is important to note that our results reflect AB-type KRAB domain configurations. Currently, it is unclear which sequence features determine whether a KRAB-A subdomain alone is sufficient to interact with TRIM28 and to mediate repressor activity. The transition from aKRAB to mKRAB occurred during the evolution of the common ancestors of coelacanth and tetrapods at a time the vertebrates adapted from living in water to living on land. 

A limited number of amino acid changes was required to transform the aKRAB-A of human PRDM9 into an mKRAB-A with canonical function in respect to TRIM28 binding and concomitant transcriptional repression activity. The changes suggested the requirement for a specific landscape of hydrophobic and charged amino acid side-chains in the context of a specific core structure consisting of three α-helices arranged in a characteristic topology. The 3-D patterns of freely accessible and correctly orientated residues pointing outwards may provide a framework for a distinct protein-protein interaction code of KRAB and TRIM28. The occurrence of the central helix-2 structure in KRAB-A models from bivalves to humans highlights this helical conformation as a building block for mediating and fine-tuning molecular interactions during evolution. Our structure-function analysis might support and inspire approaches that use KRAB domains for targeted gene regulation [76] epigenome editing [77] and synthetic biology [78].

## Figures and Tables

**Table 1:** Stratification of the ancestral KRAB-A domain. Subgroup-specific scores obtained with the different profile HMMs.

Subfamily	Group	Count	HMM hsKRAB-A	HMM PRDM9 Ortho	HMM SSX Ortho	HMM Lcha ZNF
**mKRAB-A**	hsKRAB-A	416	26.50 [3.57–31.20]	4.20 [<2–14.89]	4.31 [<2–10.57]	5.23 [<2–16.59]
**aKRAB-A**	PRDM9 ortho	183	8.59 [<2–10.20]	29.72 [13.54–31.31]	13.92 [4.52–16.55]	13.51 [3.28–14.85]
**aKRAB-A**	SSX ortho	224	5.64 [<2–7.70]	14.69 [3.64–20.89]	22.12 [14.68–24.17]	8.74 [2.74–11.23]
**aKRAB-A**	lcha ZNF	257	7.01 [4.59–10.64]	15.19 [8.48–18.31]	9.89 [6.89–13.33]	22.68 [16.51–24.14]

Median scores (−log10 HMMER E-values) [range].

**Table 2:** Stratification of the ancestral KRAB-A domain. Group-wise comparison of the scores obtained with the different profile HMMs.

Group	hsKRAB-A	PRDM9 Ortho	SSX Ortho	Lcha ZNF
hsKRAB-A	-	6.77 × 10^−132^	2.65 × 10^−132^	2.04 × 10^−129^
PRDM9 ortho	1.65 × 10^−61^	-	1.38 × 10^−59^	1.40 × 10^−60^
SSX ortho	6.85 × 10^−75^	4.18 × 10^−71^	-	6.83 × 10^−75^
lcha ZNF	1.15 × 10^-085^	2.73 × 10^−85^	1.15 × 10^−85^	-

*p*-values (Wilcoxon-Mann-Whitney, two-sided).

## Data Availability

All data generated or analyzed during this study are included in this published article [and its Supplementary Files].

## Supplementary Materials

The following supporting information can be downloaded at: https://www.mdpi.com/article/10.3390/ijms23031072/s1.

## Abbreviations

GST	Glutathione S-transferase
KO	Knockout
KRAB	Krüppel-associated box
aKRAB	Ancestral KRAB
mKRAB	Modern KRAB
KZNF	KRAB zinc finger
PRDM	PR/SET Domain
SETDB1	SET domain bifurcated histone lysine methyltransferase 1
SSX	Synovial sarcoma X breakpoint
TRIM28	Tripartite motif containing 28
ZNF	C2H2 zinc finger

## References

[1] Lambert S.A., Jolma A., Campitelli L.F., Das P.K., Yin Y., Albu M., Chen X., Taipale J., Hughes T.R., Weirauch M.T. (2018). The Human Transcription Factors. Cell.

[2] Vaquerizas J.M., Kummerfeld S.K., Teichmann S.A. (2009). Luscombe NM. A census of human transcription factors: Function, expression and evolution. Nat. Rev. Genet..

[3] Ninova M., Fejes Toth K., Aravin A.A. (2019). The control of gene expression and cell identity by H3K9 trimethylation. Development.

[4] Schlesinger S., Meshorer E. (2019). Open Chromatin, Epigenetic Plasticity, and Nuclear Organization in Pluripotency. Dev. Cell.

[5] Lee T.I., Young R.A. (2013). Transcriptional regulation and its misregulation in disease. Cell.

[6] Segert J.A., Gisselbrecht S.S., Bulyk M.L. (2021). Transcriptional Silencers: Driving Gene Expression with the Brakes On. Trends in genetics. TIG.

[7] Perissi V., Jepsen K., Glass C.K., Rosenfeld M.G. (2010). Deconstructing repression: Evolving models of co-repressor action. Nat. Rev. Genet..

[8] Ooi L., Wood I.C. (2007). Chromatin crosstalk in development and disease: Lessons from REST. Nat. Rev. Genet..

[9] Thiesen H.J. (1990). Multiple genes encoding zinc finger domains are expressed in human T cells. New Biol..

[10] Emerson R.O., Thomas J.H. (2009). Adaptive evolution in zinc finger transcription factors. PLoS Genet..

[11] Imbeault M., Helleboid P.Y., Trono D. (2017). KRAB zinc-finger proteins contribute to the evolution of gene regulatory networks. Nature.

[12] Liu H., Chang L.H., Sun Y., Lu X., Stubbs L. (2014). Deep vertebrate roots for mammalian zinc finger transcription factor subfamilies. Genome Biol. Evol..

[13] Birtle Z., Ponting C.P. (2006). Meisetz and the birth of the KRAB motif. Bioinformatics.

[14] Baker Z., Schumer M., Haba Y., Bashkirova L., Holland C., Rosenthal G.G., Przeworski M. (2017). Repeated losses of PRDM9-directed recombination despite the conservation of PRDM9 across vertebrates. eLife.

[15] Deuschle U., Meyer W.K., Thiesen H.J. (1995). Tetracycline-reversible silencing of eukaryotic promoters. Mol. Cell Biol..

[16] Ecco G., Imbeault M., Trono D. (2017). KRAB zinc finger proteins. Development.

[17] Lupo A., Cesaro E., Montano G., Zurlo D., Izzo P., Costanzo P. (2013). KRAB-Zinc Finger Proteins: A Repressor Family Displaying Multiple Biological Functions. Curr. Genom..

[18] Cheng C.T., Kuo C.Y., Ann D.K. (2014). KAPtain in charge of multiple missions: Emerging roles of KAP1. World J. Biol. Chem..

[19] Iyengar S., Farnham P.J. (2011). KAP1 protein: An enigmatic master regulator of the genome. J. Biol. Chem..

[20] Cesaro E., Lupo A., Rapuano R., Pastore A., Grosso M., Costanzo P. (2021). ZNF224 Protein: Multifaceted Functions Based on Its Molecular Partners. Molecules.

[21] Czerwinska P., Mazurek S., Wiznerowicz M. (2017). The complexity of TRIM28 contribution to cancer. J. Biomed. Sci..

[22] Sobocinska J., Molenda S., Machnik M., Oleksiewicz U. (2021). KRAB-ZFP Transcriptional Regulators Acting as Oncogenes and Tumor Suppressors: An Overview. Int. J. Mol. Sci..

[23] Thomas J.H., Schneider S. (2011). Coevolution of retroelements and tandem zinc finger genes. Genome Res..

[24] Wolf G., de Iaco A., Sun M.A., Bruno M., Tinkham M., Hoang D., Mitra A., Ralls S., Trono D., Macfarlan T.S. (2020). KRAB-zinc finger protein gene expansion in response to active retrotransposons in the murine lineage. eLife.

[25] Bruno M., Mahgoub M., Macfarlan T.S. (2019). The Arms Race Between KRAB-Zinc Finger Proteins and Endogenous Retroelements and Its Impact on Mammals. Annu. Rev. Genet..

[26] Pontis J., Planet E., Offner S., Turelli P., Duc J., Coudray A., Theunissen T.W., Jaenisch R., Trono D. (2019). Hominoid-Specific Transposable Elements and KZFPs Facilitate Human Embryonic Genome Activation and Control Transcription in Naive Human ESCs. Cell Stem. Cell.

[27] Gilbert L.A., Larson M.H., Morsut L., Liu Z., Brar G.A., Torres S.E., Stern-Ginossar N., Brandman O., Whitehead E.H., Doudna J.A. (2013). CRISPR-mediated modular RNA-guided regulation of transcription in eukaryotes. Cell.

[28] MacLeod R.S., Cawley K.M., Gubrij I., Nookaew I., Onal M., O’Brien C.A. (2019). Effective CRISPR interference of an endogenous gene via a single transgene in mice. Sci. Rep..

[29] Wang L., Wang E., Prado Balcazar J., Wu Z., Xiang K., Wang Y., Huang Q., Negrete M., Chen K.Y., Li W. (2021). Chromatin Remodeling of Colorectal Cancer Liver Metastasis is Mediated by an HGF-PU.1-DPP4 Axis. Adv. Sci..

[30] Yang Z., Li L., Turkoz A., Chen P., Harari-Steinfeld R., Bobbin M., Stefanson O., Choi H., Pietrobon V., Alphson B. (2021). Contextual reprogramming of CAR-T cells for treatment of HER2(+) cancers. J. Transl. Med..

[31] Das S., Chadwick B.P. (2021). CRISPR mediated targeting of DUX4 distal regulatory element represses DUX4 target genes dysregulated in Facioscapulohumeral muscular dystrophy. Sci. Rep..

[32] Olson A., Basukala B., Lee S., Gagne M., Wong W.W., Henderson A.J. (2020). Targeted Chromatinization and Repression of HIV-1 Provirus Transcription with Repurposed CRISPR/Cas9. Viruses.

[33] Born N., Thiesen H.J., Lorenz P. (2014). The B-subdomain of the Xenopus laevis XFIN KRAB-AB domain is responsible for its weaker transcriptional repressor activity compared to human ZNF10/Kox1. PLoS ONE.

[34] Peng H., Gibson L.C., Capili A.D., Borden K.L., Osborne M.J., Harper S.L., Speicher D.W., Zhao K., Marmorstein R., Rock T.A. (2007). The structurally disordered KRAB repression domain is incorporated into a protease resistant core upon binding to KAP-1-RBCC domain. J. Mol. Biol..

[35] Vissing H., Meyer W.K., Aagaard L., Tommerup N., Thiesen H.J. (1995). Repression of transcriptional activity by heterologous KRAB domains present in zinc finger proteins. FEBS Lett..

[36] Huntley S., Baggott D.M., Hamilton A.T., Tran-Gyamfi M., Yang S., Kim J., Gordon L., Branscomb E., Stubbs L. (2006). A comprehensive catalog of human KRAB-associated zinc finger genes: Insights into the evolutionary history of a large family of transcriptional repressors. Genome Res..

[37] Grey C., Baudat F., de Massy B. (2018). PRDM9, a driver of the genetic map. PLoS Genet..

[38] Spruce C., Dlamini S., Ananda G., Bronkema N., Tian H., Paigen K., Carter G.W., Baker C.L. (2020). HELLS and PRDM9 form a pioneer complex to open chromatin at meiotic recombination hot spots. Genes Dev..

[39] Lim F.L., Soulez M., Koczan D., Thiesen H.J., Knight J.C. (1998). A KRAB-related domain and a novel transcription repression domain in proteins encoded by SSX genes that are disrupted in human sarcomas. Oncogene.

[40] Mihola O., Trachtulec Z., Vlcek C., Schimenti J.C., Forejt J. (2009). A mouse speciation gene encodes a meiotic histone H3 methyltransferase. Science.

[41] Oliver P.L., Goodstadt L., Bayes J.J., Birtle Z., Roach K.C., Phadnis N., Beatson S.A., Lunter G., Malik H.S., Ponting C.P. (2009). Accelerated evolution of the Prdm9 speciation gene across diverse metazoan taxa. PLoS Genet..

[42] Thomas J.H., Emerson R.O., Shendure J. (2009). Extraordinary molecular evolution in the PRDM9 fertility gene. PLoS ONE.

[43] Imai Y., Baudat F., Taillepierre M., Stanzione M., Toth A., de Massy B. (2017). The PRDM9 KRAB domain is required for meiosis and involved in protein interactions. Chromosoma.

[44] Patel A., Horton J.R., Wilson G.G., Zhang X., Cheng X. (2016). Structural basis for human PRDM9 action at recombination hot spots. Genes Dev..

[45] Gure A.O., Wei I.J., Old L.J., Chen Y.T. (2002). The SSX gene family: Characterization of 9 complete genes. Int. J. Cancer.

[46] Johansen S., Gjerstorff M.F. (2020). Interaction between Polycomb and SSX Proteins in Pericentromeric Heterochromatin Function and Its Implication in Cancer. Cells.

[47] Smith H.A., McNeel D.G. (2010). The SSX family of cancer-testis antigens as target proteins for tumor therapy. Clin. Dev. Immunol..

[48] Helleboid P.Y., Heusel M., Duc J., Piot C., Thorball C.W., Coluccio A., Pontis J., Imbeault M., Turelli P., Aebersold R. (2019). The interactome of KRAB zinc finger proteins reveals the evolutionary history of their functional diversification. EMBO J..

[49] Kumar S., Stecher G., Suleski M., Hedges S.B. (2017). TimeTree: A Resource for Timelines, Timetrees, and Divergence Times. Mol. Biol. Evol..

[50] Al Chiblak M., Steinbeck F., Thiesen H.J., Lorenz P. (2019). DUF3669, a “domain of unknown function” within ZNF746 and ZNF777, oligomerizes and contributes to transcriptional repression. BMC Mol. Cell Biol..

[51] Margolin J.F., Friedman J.R., Meyer W.K., Vissing H., Thiesen H.J., Rauscher F.J. (1994). Kruppel-associated boxes are potent transcriptional repression domains. Proc. Natl. Acad. Sci. USA.

[52] Peng H., Ivanov A.V., Oh H.J., Lau Y.F., Rauscher F.J. (2009). Epigenetic gene silencing by the SRY protein is mediated by a KRAB-O protein that recruits the KAP1 co-repressor machinery. J. Biol. Chem..

[53] Witzgall R., O’Leary E., Leaf A., Onaldi D., Bonventre J.V. (1994). The Kruppel-associated box-A (KRAB-A) domain of zinc finger proteins mediates transcriptional repression. Proc. Natl. Acad. Sci. USA.

[54] Thiesen H.J. (1996). From repression domains to designer zinc finger proteins: A novel strategy of intracellular immunization against HIV. Gene Expr..

[55] Friedman J.R., Fredericks W.J., Jensen D.E., Speicher D.W., Huang X.P., Neilson E.G., Rauscher F.J. (1996). KAP-1, a novel corepressor for the highly conserved KRAB repression domain. Genes Dev..

[56] Moosmann P., Georgiev O., Le Douarin B., Bourquin J.P., Schaffner W. (1996). Transcriptional repression by RING finger protein TIF1 beta that interacts with the KRAB repressor domain of KOX1. Nucleic Acids Res..

[57] Schultz D.C., Ayyanathan K., Negorev D., Maul G.G., Rauscher F.J. (2002). SETDB1: A novel KAP-1-associated histone H3, lysine 9-specific methyltransferase that contributes to HP1-mediated silencing of euchromatic genes by KRAB zinc-finger proteins. Genes Dev..

[58] Sripathy S.P., Stevens J., Schultz D.C. (2006). The KAP1 corepressor functions to coordinate the assembly of de novo HP1-demarcated microenvironments of heterochromatin required for KRAB zinc finger protein-mediated transcriptional repression. Mol. Cell. Biol..

[59] Jumper J., Evans R., Pritzel A., Green T., Figurnov M., Ronneberger O., Tunyasuvunakool K., Bates R., Zidek A., Potapenko A. (2021). Highly accurate protein structure prediction with AlphaFold. Nature.

[60] Mirdita M., Schütze K., Moriwaki Y., Heo L., Ovchinnikov S., Steinegger M. (2021). ColabFold-Making protein folding accessible to all. bioRxiv.

[61] Stoll G.A., Oda S.I., Chong Z.S., Yu M., McLaughlin S.H., Modis Y. (2019). Structure of KAP1 tripartite motif identifies molecular interfaces required for retroelement silencing. Proc. Natl. Acad. Sci. USA.

[62] Sun Y., Keown J.R., Black M.M., Raclot C., Demarais N., Trono D., Turelli P., Goldstone D.C. (2019). A Dissection of Oligomerization by the TRIM28 Tripartite Motif and the Interaction with Members of the Krab-ZFP Family. J. Mol. Biol..

[63] Mannini R., Rivieccio V., D’Auria S., Tanfani F., Ausili A., Facchiano A., Pedone C., Grimaldi G. (2006). Structure/function of KRAB repression domains: Structural properties of KRAB modules inferred from hydrodynamic, circular dichroism, and FTIR spectroscopic analyses. Proteins.

[64] Murphy K.E., Shylo N.A., Alexander K.A., Churchill A.J., Copperman C., Garcia-Garcia M.J. (2016). The Transcriptional Repressive Activity of KRAB Zinc Finger Proteins Does Not Correlate with Their Ability to Recruit TRIM28. PLoS ONE.

[65] Staby L., O’Shea C., Willemoes M., Theisen F., Kragelund B.B., Skriver K. (2017). Eukaryotic transcription factors: Paradigms of protein intrinsic disorder. Biochem. J..

[66] Tycko J., del Rosso N., Hess G.T., Aradhana A., Banerjee A., Mukund A., Van M.V., Ego B.K., Yao D., Spees K. (2020). High-Throughput Discovery and Characterization of Human Transcriptional Effectors. Cell.

[67] Amemiya C.T., Alfoldi J., Lee A.P., Fan S., Philippe H., Maccallum I., Braasch I., Manousaki T., Schneider I., Rohner N. (2013). The African coelacanth genome provides insights into tetrapod evolution. Nature.

[68] Biscotti M.A., Adolfi M.C., Barucca M., Forconi M., Pallavicini A., Gerdol M., Canapa A., Schartl M. (2018). A Comparative View on Sex Differentiation and Gametogenesis Genes in Lungfish and Coelacanths. Genome Biol. Evol..

[69] Parvanov E.D., Tian H., Billings T., Saxl R.L., Spruce C., Aithal R., Krejci L., Paigen K., Petkov P.M. (2017). PRDM9 interactions with other proteins provide a link between recombination hotspots and the chromosomal axis in meiosis. Mol. Biol. Cell.

[70] de Bruijn D.R., dos Santos N.R., Kater-Baats E., Thijssen J., van den Berk L., Stap J., Balemans M., Schepens M., Merkx G., van Kessel A.G. (2002). The cancer-related protein SSX2 interacts with the human homologue of a Ras-like GTPase interactor, RAB3IP, and a novel nuclear protein, SSX2IP. Genes Chromosomes Cancer.

[71] Schmitges F.W., Radovani E., Najafabadi H.S., Barazandeh M., Campitelli L.F., Yin Y., Jolma A., Zhong G., Guo H., Kanagalingam T. (2016). Multiparameter functional diversity of human C2H2 zinc finger proteins. Genome Res..

[72] Biscotti M.A., Gerdol M., Canapa A., Forconi M., Olmo E., Pallavicini A., Barucca M., Schartl M. (2016). The Lungfish Transcriptome: A Glimpse into Molecular Evolution Events at the Transition from Water to Land. Sci. Rep..

[73] Wheeler T.J., Clements J., Finn R.D. (2014). Skylign: A tool for creating informative, interactive logos representing sequence alignments and profile hidden Markov models. BMC Bioinform..

[74] Kumar S., Stecher G., Li M., Knyaz C., Tamura K. (2018). MEGA X: Molecular Evolutionary Genetics Analysis across Computing Platforms. Mol. Biol. Evol..

[75] Remboutsika E., Lutz Y., Gansmuller A., Vonesch J.L., Losson R., Chambon P. (1999). The putative nuclear receptor mediator TIF1alpha is tightly associated with euchromatin. J. Cell Sci..

[76] Alerasool N., Segal D., Lee H., Taipale M. (2020). An efficient KRAB domain for CRISPRi applications in human cells. Nat. Methods.

[77] Lau C.H., Suh Y. (2018). In vivo epigenome editing and transcriptional modulation using CRISPR technology. Transgenic Res..

[78] Kim H., Bojar D., Fussenegger M. (2019). A CRISPR/Cas9-based central processing unit to program complex logic computation in human cells. Proc. Natl. Acad. Sci. USA.

