# Supplementary material for: The KRAB Domain of ZNF10 Guides the Identification of Specific Amino Acids That Transform the Ancestral KRAB-A-Related Domain Present in Human PRDM9 into a Canonical Modern KRAB-A Domain
Source: Int J Mol Sci. 2022 Jan 19;23(3):1072. doi: 10.3390/ijms23031072 (PMC8835667; doi:10.3390/ijms23031072)
Supplement: Supplementary file 1 [file ijms-23-01072-s001.zip › Figure_S2.pdf]

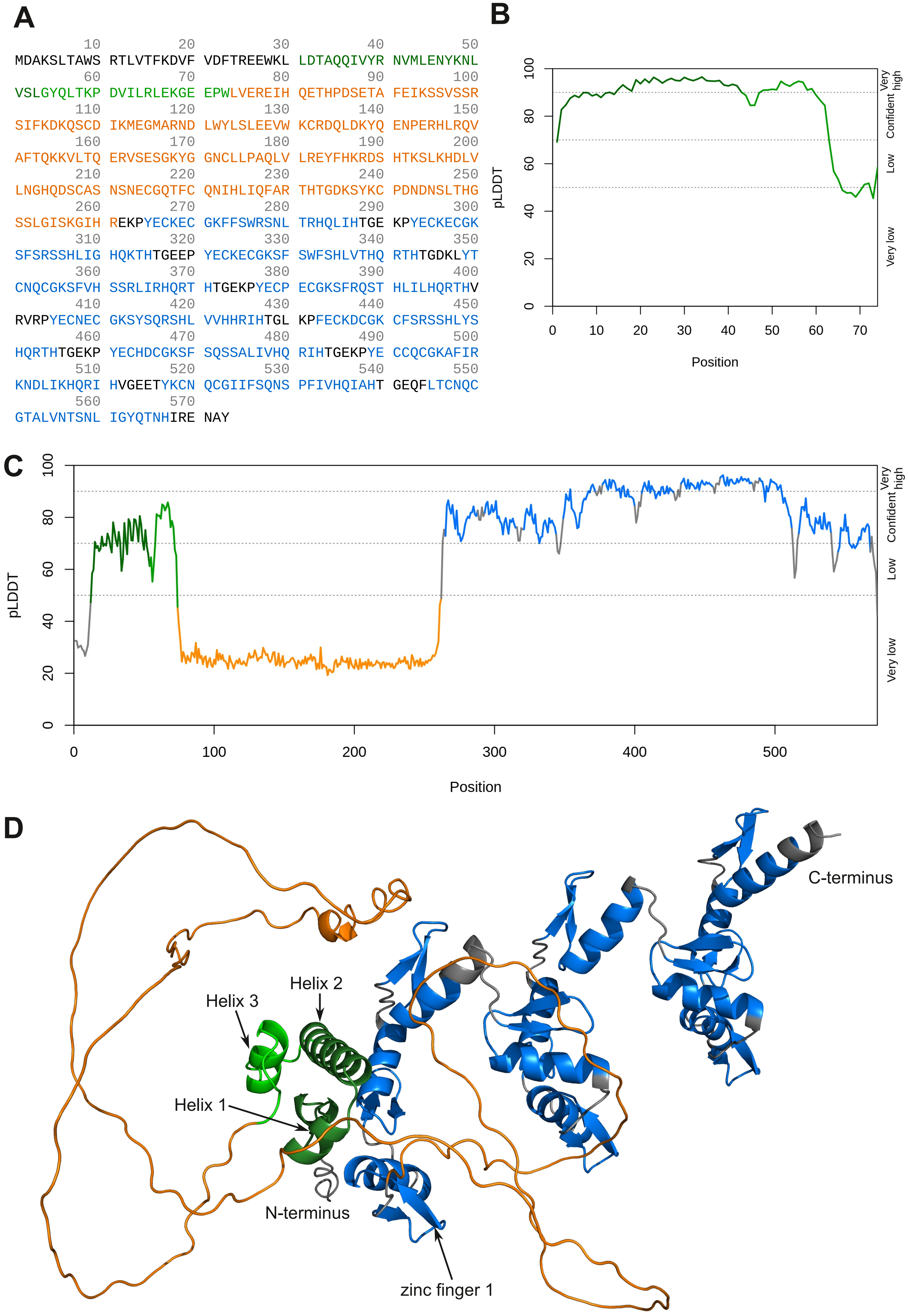
A
10 20 30 40 50
MDAKSLTAWS RTLVTFKDVF VDFTREEWKL LDTAQQIVYR NVMLENYKNL
60 70 80 90 100
VSLGYQLTKP DVILRLEKGE EPWLVEREIH QETHPDSETA FEIKSSVSSR
110 120 130 140 150
SIFKDKQSCD IKMEGMARND LWYLSLEEVW KCRDQLDKYQ ENPERHLRQV
160 170 180 190 200
AFTQKKVLTQ ERVSESGKYG GNCLLPAQLV LREYFHKRDS HTKSLKHDLV
210 220 230 240 250
LNGHQDSCAS NSNECGQTFC QNIHLIQFAR THTGDKSYKC PDNDNSLTHG
260 270 280 290 300
SSLGISKGIH REKPYECKEC GKFFSWRSNL TRHQLIHTGE KPYECKECGK
310 320 330 340 350
SFSRSSHLIG HQKTHTGEEP YECKECGKSF SWFSHLVTHQ RTHTGDKLYT
360 370 380 390 400
CNQCGKSFVH SSRLIRHQRT HTGEKPYECP ECGKSFRQST HLILHQRTHV
410 420 430 440 450
RVRPYECNEC GKSYSQRSHL VVHHRIHTGL KPFECKDCGK CFSRSSHLYS
460 470 480 490 500
HQRTHTGEKP YECHDCGKSF SQSSALIVHQ RIHTGEKPYE CCQCGKAFIR
510 520 530 540 550
KNDLIKHQRI HVGEETYKCN QCGIIFSQNS PFIVHQIAHT GEQFLTCNQC
560 570
GTALVNTSNL IGYQTNHIRE NAY
B
pLDDT
Position
Very high
Confident
Low
Very low
C
pLDDT
Position
Very high
Confident
Low
Very low
D
Helix 1
Helix 2
Helix 3
N-terminus
C-terminus
zinc finger 1
