# Supplementary material for: The KRAB Domain of ZNF10 Guides the Identification of Specific Amino Acids That Transform the Ancestral KRAB-A-Related Domain Present in Human PRDM9 into a Canonical Modern KRAB-A Domain
Source: Int J Mol Sci. 2022 Jan 19;23(3):1072. doi: 10.3390/ijms23031072 (PMC8835667; doi:10.3390/ijms23031072)
Supplement: Supplementary file 1 [file ijms-23-01072-s001.zip › ijms-1537523 Supplementary Files.pdf]

Supplementary Files

Figure S1. Test of expression for the various Gal4-KRAB fusion proteins used in reporter assays. Results from two experiments. HeLa cells were transfected with 2µg of the indicated constructs. Aliquots of total cell extracts made with 1 x Lämmli-type SDS sample buffer 24 hours post-transfection were loaded onto a 12% SDS-PAGE gel, blotted and probed with anti-Gal4 antibodies (green signals) and anti-GAPDH antibodies (red signals). The Gal4 DNA binding domain (Gal4-DBD) was visualized with rabbit anti-Gal4 (at 0.2µg/ml; sc-577, Santa Cruz Biotechnology) and goat anti-rabbit IgG-IRDye-800CW (Li-Cor #926-32211). GAPDH was detected with mouse monoclonal antibodies (at 0.08µg/ml; Millipore #MAP374) and the secondary anti-mouse IgG antibody indicated in the Methods section.

Figure S2. AlphaFold2 confidence plots and 3D model of full-length human ZNF10.
Color code: Green (KRAB-AB;(subdomain A = dark green, subdomain A = light green); Orange (unstructured spacer region between KRAB and zinc finger array); Blue (canonical C2H2 zinc finger motifs); Black (none of the above).
A. Amino acid sequence of human KZNF protein ZNF10/KOX1 (Uniprot P21506)
B. Plotted confidence score per residue (pLDDT, see Jumper2021 [59]) of the 3D model obtained for human ZNF10 KRAB-AB (respective 3D model shown in Figure 6B as well as in Figure 7A)
C. Plotted confidence score per residue (pLDDT, see Jumper2021 [59]) of the 3D model of full-length ZNF10 contained in the Alphafold2 database at https://alphafold.ebi.ac.uk/entry/P21506
D. 3D model of full-length ZNF10 obtained from the AlphaFold2 database and colored according to domain.

Table S1. Compilation of the ancestral KRAB-A encoding genes in all species. The workflow is described in the Methods section.

Table S2.
Long supplement version of Figure 9: List and frequency distributions of amino acid residues in ancestral and modern KRAB domains. Amino acid frequencies of five KRAB proteins representing blueprints for each specific group include human ZNF10 KRAB-AB (residues 13-85) as a mKRAB-AB, human SSX1/22-97 for the SSX aKRAB subgroup, human PRDM9(24-97) to represent the PRDM9 aKRAB subgroup and KZNF4712/59-134 for the coelacanth aKRAB group. Also included is the mussel (mgal = *Mytilus galloprovincialis*) PRDM9/80-152 (Uniprot A0A3L5TRV6) as the aKRAB with the deepest evolutionary root. Upper part: The distribution of each shown residue of a blueprint was scored against the groups of human mKRAB, the PRDM9, SSX and coelacanth KZNF aKRAB sequences by counting their frequencies. Note, that counting of amino acids in sequences following KRAB-A was done irrespectively of the existence of an actual conserved KRAB-B. Frequencies of individual AA present in these KRAB sequences have been visualized in logos presented in Figure 1. In case of human mKRAB, 399 human mKRAB proteins are used instead of 416 as in Figure 1. Amino acid counts are given for positions A02 to A42 and B01 to B32. Note, that position A32 is only present in modern KRAB structures but not in ancestral KRAB structures. The ZNF10 sequence of the KRAB domain initially discovered based on heptad-repeats-of-leucines MLLL (within A32 to B11;

Thiesen1990 [9];) in resemblance to leucine zipper structures is marked in green. The sequences that distinguish mutant KRAB-A constructs of PRDM9 are listed in Figure 3A and are visualized in part in Figure 6, Figure 7 and Figure 8. The PDB files of all mutants are in the Supplement. Positions marked in orange are common to mKRAB as well as aKRAB and show residues whose side chains point outward from the core of the KRAB body due to the alignment. Putative amino acid contact sites that are supposed to be utilized by aKRAB from KZNF of the coelacanth are emphasized in yellow. The current presentation in Figure 9 and in Table S2 evinces amino acids to be contacted by modern human TRIM28 opposed to ancestral TRIM28 binding in the coelacanth. Note that modern TRIM28 is adapted to recognize modern 3D-KRAB-AB configurations presented by ZNF10 and ancestral TRIM28 amino acid 3D configuration presented by aKRAB-ZNF proteins in the coelacanth. In case an aKRAB configuration is chosen for aligning KRAB amino acid sequences by closing the gap at A32, the calculation of amino acid abundancies has to be redone accordingly. Amino acids assumed to be relevant for shaping the structure of the B domain of ZNF 10 are shown in pink. The amino acids from A02 to B32 are predicted to selectively interact either intramolecularly within the KRAB domain, with other parts of the cognate protein or with proteins that directly bind KRAB domains. In the latter case, amino acid specificities might correlate with phylogeny.

Lower part: KRAB sequences of ZNF10, PRDM9 and lcha4712 are compared to ZNF746b (AlChiblak2019, [50]. Here, AA positions presenting AA of ZNF10 are interrogated whose substitution by all other 19 amino acids led to an abrogation of greater than 10-fold (data from (Tycko2020 [66]). Here, only the numbers of different amino acids are listed that effect the ZNF10 mediated repression in vivo by more than 10-fold. Furthermore, the amino acid positions listed show a loss of repressor activity due to the replacement by proline. AA positions and type of mutants generated by Margolin et al., (Margolin1994, [51]) are indicated as well.

PDBs.zip

3D structures obtained by AlphaFold2 modelling in PDB file format

HMMs.zip

HMM v2 and HMM v3 matrices for interrogating protein sequences using HMMER version 2 or 3.
