# Supplementary figures and images for: The KRAB Domain of ZNF10 Guides the Identification of Specific Amino Acids That Transform the Ancestral KRAB-A-Related Domain Present in Human PRDM9 into a Canonical Modern KRAB-A Domain
Source: Int J Mol Sci. 2022 Jan 19;23(3):1072. doi: 10.3390/ijms23031072 (PMC8835667; doi:10.3390/ijms23031072)

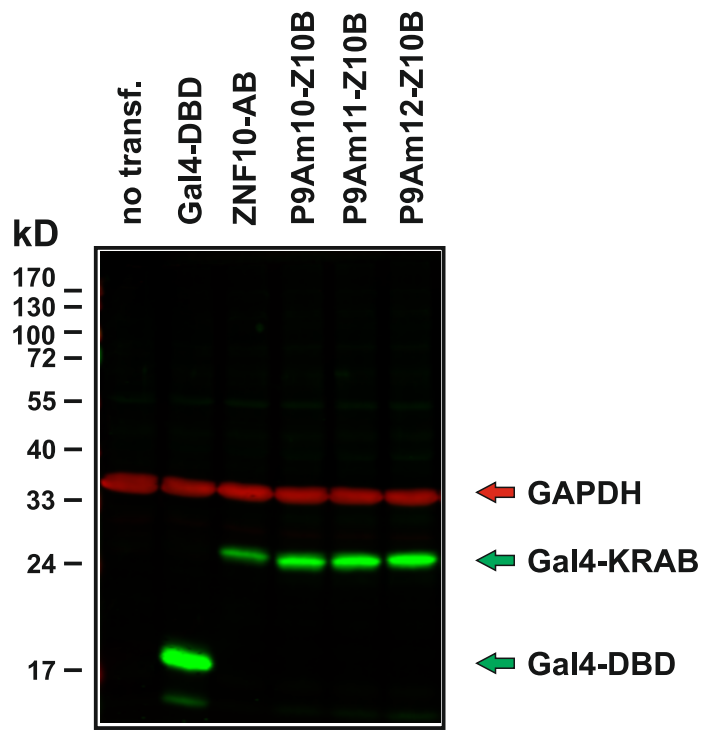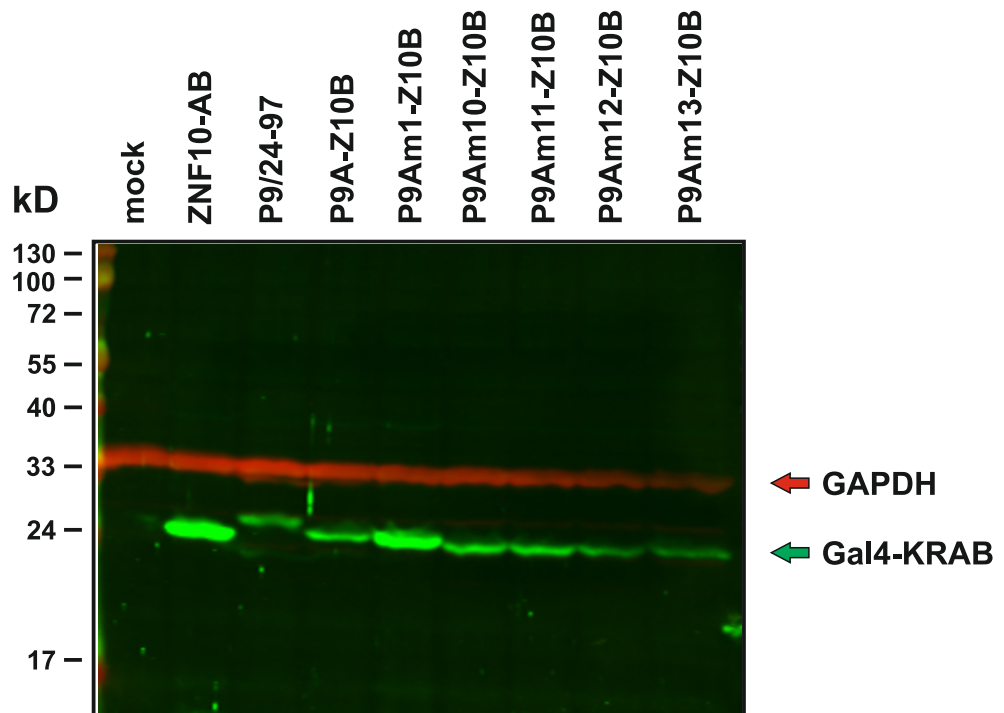

Supplement: Supplementary file 1 [file ijms-23-01072-s001.zip › Figure_S1.pdf]
